# Influence of the Saccharide Structure on Cargo Loading, Thermal Properties, and Lectin Binding of Amphiphilic Glycopolymer-Polylactic Acid Block Copolymer Nanoparticles

**DOI:** 10.1021/acs.bioconjchem.5c00217

**Published:** 2025-07-17

**Authors:** Kevin A. Green, Anuja S. Kulkarni, Penelope E. Jankoski, Rachel M. Worden, Bayleigh M. Loving, Blaine Derbigny, Tristan D. Clemons, Davita L. Watkins, Sarah E. Morgan

**Affiliations:** 1 School of Polymer Science and Engineering, 5104 The University of Southern Mississippi, Hattiesburg, Mississippi 39406, United States; 2 Department of Chemistry & Biochemistry, 2647 The Ohio State University, Columbus, Ohio 43210, United States; 3 William G. Lowrie Department of Chemical and Biomolecular Engineering, 2647 The Ohio State University, 151 W Woodruff Ave., Columbus, Ohio 43210, United States; 4 Department of Chemistry and Biochemistry, The Georgia Institute of Technology, Atlanta, Georgia 30318, United States

## Abstract

Stereospecific arrangements of saccharide molecules control biological recognition and binding with proteins. These properties can also be utilized in the design of biomaterials for applications such as polymeric drug delivery, where saccharides may enhance the ability to target specific cells. Glycopolymer block copolymers incorporating pendant saccharides at high concentration have potential for use in applications; however, there is a need for further evaluation of their structure–property relationships. Accordingly, noncytotoxic amphiphilic, hybrid block copolymers (HBCs), synthesized by coupling branched polylactic acid (PLA) with linear polyacrylamides containing hydroxyethyl, β-d-glucose, or β-d-galactose moieties, were studied to determine the influence of the stereochemistry and structure of the pendant saccharide on nanoparticle formation, cargo loading, and lectin binding properties. HBCs were prepared at a target 50:50 PLA/hydrophilic block content; all compositions yielded similar spherical nanoparticle morphologies with comparable diameters on nanoprecipitation. Thermal properties and hydrophilic dye loading levels, however, were dependent on the pendant saccharide structure, attributed to differences in intramolecular interactions in the glycopolymer blocks. These findings demonstrate the importance of understanding the structure-dependent behavior for designing HBC-based therapies.

## Graphical abstract

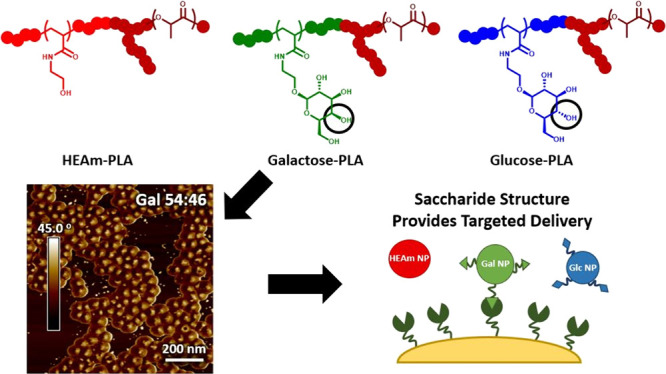

## Introduction

The development of innovative polymeric materials for biomedical applications is a rapidly advancing field, driven by the need for versatile, noncytotoxic, and stable nanocarriers for drug delivery systems.^1–3^ Hybrid block copolymers (HBCs), consisting of hydrophilic linear and hydrophobic branched blocks, have emerged as a promising class of materials due to their unique structural characteristics.^4–7^ Branched structures have shown promise for increased encapsulation of hydrophobic guest molecules due to additional avenues of encapsulation in interior void spaces, at branching sites, or through the incorporation of additional secondary interactions.^5,8^ HBCs, like traditional amphiphilic block copolymers, self-assemble into well-defined nanostructures that serve as versatile platforms for targeted therapy and diagnostic applications. Solvent exchange methods are often employed to induce block copolymer nanoprecipitation and formation of polymeric nanostructures.^9^ The shape, size, and stability of the nanostructures depend on multiple factors, including rate of mixing,^10,11^ solvent composition and order of addition,^12,13^ and block copolymer molecular weight, branching, and hydrophilic/hydrophobic balance (HHB).^14,15^ Highly stable systems can be obtained and used to encapsulate drugs that protect them from degradation and improve their bioavailability.^16–18^ However, traditional hydrophilic blocks, like poly­(ethylene glycol) (PEG), have lately been associated with several drawbacks regarding their biocompatibility and bioavailability.^19–21^

A recent shift to the use of natural materials for the fabrication of drug-delivery vehicles has garnered significant attention.^22^ The integration of saccharide-modified polymers (i.e., glycopolymers) into therapeutic delivery vehicles has shown great promise due to their biocompatibility, high water solubility, and natural cell targeting capabilities.^23–27^ Saccharides and glycopolymers with pendant saccharides have multivalent interactions used to mimic the “glycocluster” effect.^28^ Multivalency refers to the simultaneous interaction of multiple binding sites on one molecule with multiple ligands on another, significantly increasing the overall binding affinity.^29,30^ Human lectins are proteins that bind to carbohydrates found throughout the body.^31^ These materials are capable of recognizing saccharides based on the patterns associated with their stereochemistry.^32,33^ Sun et al. recently highlighted the selectivity of lectin-carbohydrate interactions by investigating the binding affinity of glyco-nanoparticles with different stereoisomer saccharides (α-glucose, β-glucose, α-mannose, and α-galactose) and the lectin Concanavalin A (Con A).^32^ They reported that both α-glucose and α-mannose were capable of binding to Con A, whereas β-glucose and α-galactose did not. Gou et al. demonstrated the layer-by-layer assembly of lectins with glycopolymers, using Con A and peanut agglutinin (PNA), a plant lectin that binds specifically with galactose, and mannose- and galactose-containing glycopolymers.^34^ They reported tuning the composition of the glycopolymer layers to optimize binding interactions within the multilayer bioactive films. Gou et al. highlighted the challenges associated with varying saccharide groups as differences in saccharide stereochemistry may impact chain flexibility and performance.^35^ Some lectins, in particular galectins that are receptors for β-galactose, appear prominently at the surface of malignant cells in the stomach and liver, indicating their potential use in tumor targeting.^22^ Collectively, these reports highlight the potential of glycopolymers in the design of targeted therapeutics and the importance of controlling saccharide stereochemistry for specific cell receptor binding. They also underscore the need to better understand the relationships between glycopolymer structure and its impact on lectin binding and therapeutic delivery properties.

We previously reported the synthesis and nanoparticle formation of HBCs comprised of a branched poly­(lactic acid) (PLA) block with linear β-d-glucose glycopolymer blocks of varying molecular weight.^36^ In this study, HBCs with similar HHB but varying hydrophilic block structures are prepared, and their nanoparticle formation, thermal properties, cytotoxicity, dye loading and release performance, and lectin binding properties evaluated. Thiol–ene coupling reactions are employed to prepare copolymers from starting blocks of defined molecular weight and low dispersity (Scheme 1). We report novel PLA-acrylamide block copolymers with β-d-galactose pendant groups and compare their behavior with systems containing hydroxyethyl and β-d-glucose pendant groups. The study is designed to provide an understanding of structure–property relationships critical in designing polymeric vehicles for therapeutic delivery.

**Scheme 1:**
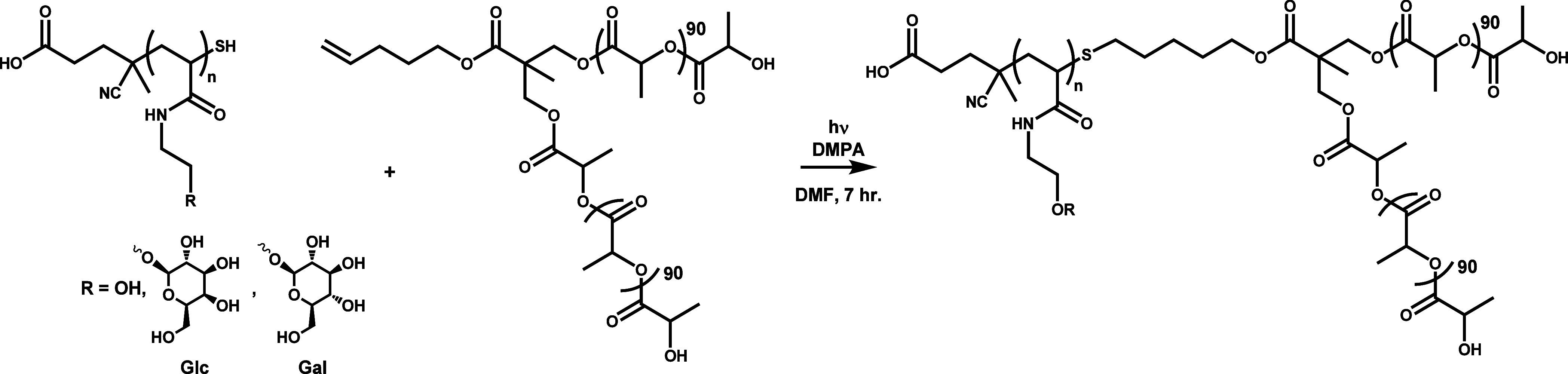
HBC Synthesis Using a Photoinitiated Thiol–Ene Click Reaction to Couple a Hydrophilic Polyacrylamide Block (pHEAm, *n* = 103, pGlcEAm, *n* = 56, and pGalEAm, *n* = 58) and a Hydrophobic-Branched PLA Block

## Experimental Methods

### Materials

*N*-Hydroxyethyl acrylamide (97%), silver trifluoromethanesulfonate (AgOTf, ≥99%), acetobromo-α-d-glucose (AcBrGlc, ≥95%), acetobromo-α-d-galactose (AcBrGal, ≥95%), 4 Å molecular sieves (powdered), anhydrous sodium sulfate (≥99%), 4′-azobis­(4-cyanovaleric acid) (V-501), trimesic acid (95%), anhydrous dimethyl sulfoxide (≥99.9%), sodium methoxide solution (25 wt % in methanol), tris hydrochloride (tris HCl, ≥99%), tris base (≥99%), 4-penten-1-ol, 2-(hydroxymethyl)-2-methylpropanoic acid, dimethyl amino pyridine (DMAP), para toluene sulfonic acid (pTSA), 1,8-diazabicyclo[5.4.0]­undec-7-ene (DBU), DOWEX 50 W XB, Amberlyst A 21 free base, 2,2-dimethoxy-2-phenylacetophenone (DMPA, ≥99%), curcumin, methyl orange (MO), 12-mercaptododecanoic acid (MDA), *N*-hydroxy-succinimide (NHS, ≥98%), ethanolamine hydrochloride (≥99%), magnesium chloride (powder, <200 μm), manganese­(II) chloride (powder and chunks, ≥99%), calcium chloride (purum, granulated, ≥97%), and lectin from *Arachis hypogaea* (peanut, PNA, lyophilized powder) were purchased from Sigma-Aldrich. Dichloromethane, ethyl acetate, hexane, methanol, tetrahydrofuran, concentrated hydrochloric acid, *N*,*N*-dimethylformamide (DMF), HPLC water, sodium azide (≥99%), ammonium hydroxide, sodium chloride, 1 M HEPES buffer solution (pH 7.3), and 1-[3-(dimethylamino)­propyl]-3-ethyl carbodiimide (EDC) were purchased from Fisher Scientific. The chain transfer agent, 4-cyano-4-(((ethylthio)­carbonothioyl)­thio)­pentanoic acid (CEP), was purchased from AmBeed. Chloroform-*d* (D, 99.9%), deuterium oxide (D, 99.9%), dimethyl sulfoxide-*d*_6_ (D, 99.9%), and *N,N*-dimethylformamide-*d*_7_ (D, 99.9%) were purchased from Cambridge Isotope Laboratories, Inc. Dicyclohexyl carbodiimide (DCC) was procured from Tokyo Chemical Industry (TCI). Hydrogen peroxide, 30% aqueous solution was purchased from Lab Alley. The LIVE/DEAD Cell Imaging Kit (488/570, Invitrogen) was obtained from Thermoscientific.

### Synthesis of Glycomonomer

The acetyl-protected glucose pendant acrylamide monomer, 2′-acrylamidoethyl-2,3,4,6-tetra-O-acetyl-β-d-glucopyranoside (AcGlcEAm) and acetyl-protected galactose pendant acrylamide monomer, 2′-acrylamidoethyl-2,3,4,6-tetra-O-acetyl-β-d-galactopyranoside (AcGalEAm), were synthesized following previous literature procedures as shown in Scheme S1 and Figure S1, respectively.^33,37,38^

### RAFT Polymerization of Hydrophilic Monomers

Hydrophilic polyacrylamides (pHEAm, pGlcEAm, and pGalEAm) were synthesized following a previously reported procedure, except that the reaction was stopped at 60% rather than 70% conversion.^38^ The synthetic procedure and reaction monitoring are summarized in Scheme S2 and Figures S2–S4 **.**

### Branched Polylactic Acid Synthesis

Hydrophobic branched PLA structures were synthesized following previous literature procedures outlined in Schemes S3–S6 and Figures S5–S8.^36^

### Linear Branched Hybrid Block Copolymer Synthesis

HBC structures were synthesized following previous literature procedures,^36^ with the exception of utilizing different hydrophilic polyacrylamides: pHEAm, pGlcEAm, and pGalEAm. The synthetic procedure is summarized in the Supporting Information, with an outline of the reaction shown in Scheme 1, and NMR spectra shown in Figures S9 and S10.

### Nanoprecipitation to Form Nanoparticles

HBCs with similar weight ratios were formed into nanoparticles via nanoprecipitation following published procedures.^4–6^ HBC samples (1 mg) were added to glass vials, followed by THF (200 μL), and the sample was vortexed until fully dissolved. The resulting solution (organic phase) was then added dropwise into a second vial containing DI water (2 mL) while vigorously stirring. The solution was then covered and allowed to equilibrate overnight, allowing residual THF to evaporate. Nanoparticle concentration was maintained at 0.5 mg mL^–1^ for characterization.

### Nuclear Magnetic Resonance (NMR) Spectroscopy

^1^H NMR spectroscopy was performed using a 400 MHz Bruker AvanceNEO spectrometer (TopSpin 4.1.3). Monomer spectra were acquired with 64 coadded scans and a delay time of 5 s. Polymer and HBC spectra were acquired with 64 coadded scans and a delay time of 2 s. All spectra were obtained using the appropriate deuterated solvents (CDCl_3_, DMSO-*d*_6_, D_2_O, or DMF-*d*_7_) as described in the figure captions and were processed and analyzed using MNova software.

### Ultraviolet-Visible (UV–Vis) Spectroscopy

Cleavage of the trithiocarbonate end group was verified using a multimode microplate reader (BioTek Synergy H1, Agilent Technologies Inc.) by monitoring the absorbance at 310 nm. Each measurement was conducted in DMSO using a sample volume of 200 μL and a sample concentration of 2.5 mg mL^–1^ at 25 °C. Absorbance data was analyzed using BioTek Gen6 data analysis software.

### Gel Permeation Chromatography with Multi-Angle Laser Light Scattering (GPC-MALLS)

Glycopolymer number-average molecular weight (*M*_n_), weight-average molecular weight (*M*_w_), and polymer dispersity (Đ) were determined using aqueous gel permeation chromatography with multiangle laser light scattering (GPC-MALLS) on an Agilent 1260 Infinity II LC system equipped with a PL Aquagel–OH 30 column (particle size 8 μm), a DAWN HELEOS-II light scattering detector (λ = 633 nm, Wyatt Technology Inc.) and an Optilab T-rEX refractometer (Wyatt Technology Inc.). TRIS buffer pH 8.0 with 0.01% (w/v) NaN_3_ was used as the eluent at a flow rate of 0.5 mL min^–1^ with a sample concentration of 20 mg mL^–1^ and an injection volume of 100 μL. The polymer refractive index increments (d*n*/d*c*) were determined using an offline refractometer (AR200 Refractometer, Reichert) at 25 °C. Wyatt ADTRA GPC/LS software (version 7.1.4.8) was used to determine the *M*_n_, *M*_w_, and Đ.

PLA molecular weights were determined using GPC-MALLS on a Waters Alliance 2695 separations module, an online MALLS detector fitted with a gallium arsenide laser, an interferometric refractometer operating at 35 °C and 685 nm, and two Agilent PLgel-mixed D columns (pore size range 50–103 Å, 3 μm bead size). Distilled THF served as the mobile phase and was delivered at a flow rate of 1.0 mL min^–1^. The absolute molecular weights were determined by MALLS using d*n*/d*c* values calculated from the refractive index detector response and assuming 100% mass recovery from the columns.

### Atomic Force Microscopy (AFM)

The nanoparticle morphology was characterized by atomic force microscopy (AFM) in Peak-Force Tapping mode (Dimension Icon AFM, Bruker) using an RTESPA-300 (*f*_0_ = 300 kHz, *k* = 40 N m^–1^, Bruker) probe. Samples were prepared by drop casting 150 μL of the nanoparticle solution onto a freshly cleaved mica substrate, waiting 30 min, wicking away excess solution, and allowing the samples to dry ambiently. Images were analyzed using NanoScope Analysis 3.00 software. Analysis of the diameters of the nanoparticles was performed in tapping mode using line width measurements in NanoScope Analysis 3.00 software following previously published procedures.^39,40^

### Dynamic Light Scattering (DLS)

Intensity average nanoparticle size measurements were conducted using a Zetasizer Nano ZS (Malvern Instrument) at 25 °C. The nanoparticle concentration was 0.5 mg mL^–1^, all measurements were performed in triplicate, and the data was analyzed using the provided Zetasizer software.

### Attenuated Total Reflectance Fourier Transform Infrared (ATR-FTIR) Spectroscopy

Room temperature ATR-FTIR spectra of dried homopolymer powders were obtained using a PerkinElmer Frontier spectrometer equipped with a Universal ATR sampling accessory. IR data were recorded with 64 scans accumulated for each spectrum. Spectra were analyzed using Spectrum IR software and normalized to the methyl peak (C–H) at 1450 cm^–1^.

### HBC Thermal Stability

Thermogravimetric analysis (TGA) and differential scanning calorimetry (DSC) were used to assess the thermal stability of the homopolymers and HBCs. A TA Instruments TGA550 was used to collect TGA thermograms. Dry powder samples were loaded onto high-temperature platinum pans (∼10 mg) and were subjected to a temperature ramp from room temperature to 800 °C at 20 °C min^–1^ under nitrogen after being equilibrated at 100 °C for 30 min to allow evolution of residual water. Afterward, the weight was normalized and the thermal degradation temperature (*T*_d,5%_) determined based on 5% sample weight loss. Thermograms were collected in triplicate. A TA Instruments DSC2500 was then used to collect DSC spectra in triplicate. Dry powder samples were loaded into Tzero hermitic aluminum pans (∼5 mg) and subjected to the following heat/cool/heat cycle performed under a N_2_ atmosphere: −50 to 200 °C by 10 °C min^–1^, cooling to −90 °C by 5 °C min^–1^, then heating to 200 °C by 10 °C min^–1^. All spectra were analyzed using TA Instruments Trios (v5.1.1.46572).

### Encapsulation Studies

Dye encapsulation procedures were performed following previously reported procedures.^36^ Structures and absorbance profiles for the dyes are shown in Figure S11. In short, for hydrophobic dye, 200 μL of a 5 mg mL^–1^ curcumin (logP value of 3)^41^ in THF solution was used as the organic phase to dissolve 1 mg HBC. The organic phase was then added dropwise to 2 mL of DI water and allowed to equilibrate overnight. Solutions were then filtered to remove unencapsulated dye that had precipitated from solution, freeze-dried, and the resulting powder was redissolved in THF for analysis. For hydrophilic dye, methyl orange (logP value of 0.15),^42^ 200 μL of THF was used as the organic phase to dissolve 1 mg HBC. The organic phase was then added dropwise to 2 mL of a 1 mg mL^–1^ of MO in DI water. Solutions were then dialyzed to remove unencapsulated and analyzed. UV–vis absorbance of respective dyes was used to determine the dye loading content (DL%) and encapsulation efficiency (EE%) of all formulations. Statistical significance was analyzed using a *t* test to compare means of the different populations. Significance is reported as the p value.

### Quartz Crystal Microbalance with Dissipation (QCM-D)

QCM-D is a highly sensitive technique that measures mass changes in real-time, thereby providing insights into the binding interactions and viscoelastic properties of absorbed layers.^43^ To determine binding profiles of nanoparticles having surface saccharides, QCM-D experiments were performed using a Q-Sense E4 with four parallel modules and an IPC Ismatec pump.

Sensors were prepared using a modified literature procedure.^34^ Gold coated quartz sensors (Biolin Scientific, QSX 301) were first prepared using a Bioforce Nanosciences UV Ozone Cleaner for 30 min, cleaned with a mixture of 35% ammonium hydroxide, 33% hydrogen peroxide, and DI water (1:1:5, v/v) for 10 min, then rinsed with DI water, and dried under nitrogen. Upon drying, the sensors were immersed into a 1 mM MDA in ethanol solution and kept overnight at room temperature to expose the thiol self-assembled monolayer on the sensors’ surface. The gold sensors were then rinsed with ethanol and DI water and then immersed into a 0.4 M EDC and 0.1 M NHS in water solution for 30 min to activate the carboxylic acid. Afterward, the sensors were rinsed with DI water, dried under nitrogen, and mounted into the QCM-D modules.

HBS buffer (10 mM HEPES buffer, 150 mM NaCl, pH 7.4) containing 1 mM of metal ions (Mg^2+^, Mn^2+^, and Ca^2+^) was prepared and used as the mobile phase for all QCM-D solutions. HBS buffer was flowed through the system at 50 μL/min and 25 °C until a flat baseline was achieved for both frequency and dissipation. Following the HBS buffer baseline, a 0.5 mg/mL lectin in HBS buffer solution was flowed over the QCM-D sensors, again until a flat baseline was achieved (about 1 h). HBS buffer was flowed afterward to remove any unbound lectin from the surface. Once stable, an ethanolamine HCl (1 M in HBS, pH 8.5) solution was used to block the unreacted NHS groups remaining on the surface to prevent their reaction with the nanoparticles. A final HBS buffer baseline was secured prior to flowing a 0.25 mg/mL solution of nanoparticles in HBS buffer. HBS buffer was flowed after the nanoparticle solution baseline was secured to remove any unbound materials. The changes in frequency and dissipation were monitored using the QTools software and analyzed using Origin64 software. The mass of material deposited on the sensor surface was calculated using eq 1.

$$\Delta m=\frac{C}{n}\Delta f$$ 1

Where Δ*m* is the mass change, *C* is the mass sensitivity constant equal to 17.7 ng cm^–2^ Hz^1–^, *n* is the harmonic overtone, and Δ*f* is the change in frequency.

### Cell Culture

Human Embryonic Kidney cells (HEK 293 cells, ATCC) were cultured in Dulbecco’s modified eagle medium (DMEM), supplemented with 10% fetal bovine serum and 1% penicillin-streptomycin at 37 °C and 5% CO_2_. Cell viability and cytotoxicity was determined using Live/Dead staining and a lactate dehydrogenase assay (LDH assay) as we have previously reported.^36^ Briefly, cells at 90% confluence were trypsinized (0.25% trypsin EDTA, 5 min) to dissociate cells, and collected into a falcon tube and centrifuged at 5000 rpm for 5 min to pellet. HEK293 cells were resuspended in supplemented DMEM and counted using a hemocytometer to determine cell concentration. Cells were diluted with additional media to a working concentration of 1 × 10^5^ cells/mL and seeded in a 96 well plate (100 μL per well). Seeded cells were left to adhere for 24 h in an incubator at 37 °C and 5% CO_2_. After 24 h, LDBC stock solutions were added to the wells to achieve the desired final concentration with nuclease free water used as a negative control, and Triton X-100 used as positive control for 100% cytotoxicity (i.e maximum LDH release). Plates were then incubated for 24 h at 37 °C and 5% CO_2_. Cytotoxicity was evaluated using the CyQUANT LDH Kit (Invitrogen) following manufacturer protocols. A Biotek Synergy microplate reader was used to assess the absorbance at 490 nm with a reference wavelength of 690 nm.

The LIVE/DEAD Cell Imaging Kit (Invitrogen) was used to assess cell viability at the highest concentration of LDBC treatment following the manufacturers protocols. Cells were plated and treated as described above. Following 24-h incubation with LDBC treatments, 70 μL of media was removed from each well, leaving 30 μL and an equal volume of freshly prepared Live/Dead (Calcein-AM and BOBO-3) stock solution added to each well. This was left to incubate for 15 min at room temperature in the dark. Five representative images were then collected per treatment using a Leica DM IL LED Fluoro SE inverted fluorescent microscope.

## Results and Discussion

### Hybrid Block Copolymer Synthesis

Block copolymers were synthesized using a thiol–ene click reaction to couple a linear hydrophilic polyacrylamide (PA) with a branched PLA, following previously reported procedures detailed in the Supporting Information and outlined in Scheme 1.^36^ The hydrophilic PAs and PLA were synthesized separately to create well-defined systems. Hydrophilic PAs (pHEAm, pGlcEAm, and pGalEAm) were synthesized using RAFT polymerization techniques outlined in Scheme S2. The PLA was synthesized via ring opening polymerization as outlined in Scheme S6, with *M*_n_ of 13.8 kDa. The target degree of polymerization (DP) of the linear hydrophilic PAs was selected to achieve an HHB ratio of 50:50 when combined with the branched PLA of constant molecular weight.

Triplicate hydrophilic PA synthesis reactions were performed to examine the polymerization kinetics and establish suitable reaction conditions. The pHEAm PA had the shortest initial induction period (51 min), followed by pGlcEAm (60 min), and pGalEAm (84 min). All three systems displayed pseudo-first-order polymerization kinetics, shown in Figure S2, and the polymerization rates followed trends similar to those observed for induction periods. Target conversions were achieved around 120, 140, and 250 min for pHEAm, pGlcEAm, and pGalEAm, respectively. After obtaining the desired molecular weights for the protected PAs, the acetyl-protecting groups were removed with simultaneous cleavage of the trithiocarbonate end group, as described in the Supporting Information. ^1^H NMR analyses of the protected monomer, protected polymer, and deprotected polymer for each of the three PAs are shown in Figure S3, and UV–vis absorbance spectra are given in Figure S4, similar to work presented in our previous report.^36^ Molecular weights and dispersity values obtained via GPC-MALLS for hydrophilic PAs are summarized in Table 1. Target molecular weights and low dispersities, Đ < 1.1, were achieved for the hydrophilic PAs. Representative GPC traces for the hydrophilic PAs and PLA are presented in Figure 1, with narrow and symmetric chromatograms observed for the polymers. Low-intensity, high-molecular-weight shoulders are observed in some of the light scattering traces, but they are not present in the corresponding refractive index traces. The shoulders are attributed to low concentrations of high-molecular-weight aggregates, commonly reported for glycopolymer solutions and other hydrophilic polymers that are capable of high levels of hydrogen bonding.^33,37,38,44^

**Figure 1:**
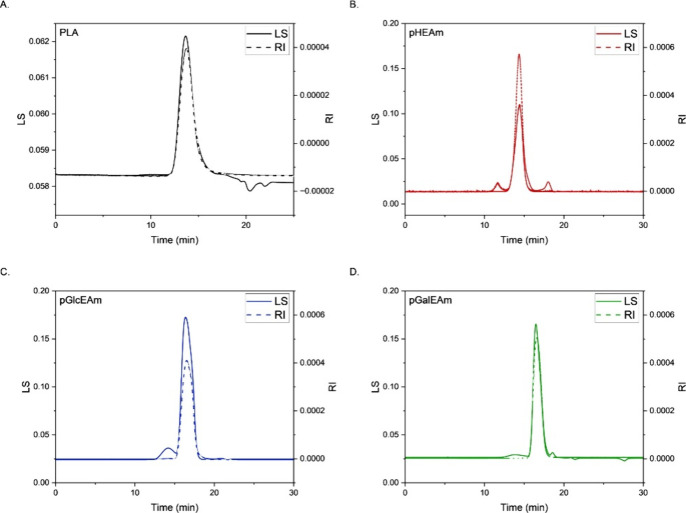
GPC trace of (A) branched PLA determined in distilled THF with two Agilent PLgel-mixed D columns (pore size range 50–103 Å). GPC-MALLS traces of (B) pHEAm, (C) pGlcEAm, and (D) pGalEAm determined in TRIS buffer (pH 8) containing 0.01% (w/v) NaN_3_ with an Aquagel–OH 30 column. Narrow and symmetric chromatograms are obtained with low levels of aggregation observed in the RI trace of the hydrophilic polymers.

**Table 1:** Composition, Conversion (ρ), Number Average Molecular Weight (*M*_n_), Dispersity (Đ), d*n*/d*c*, and Block Copolymer Naming Convention

polymer	ρ^a^	DP^a^	Mn^a^ (kDa)	Mn^b^ (kDa)	Đ^b^	d*n*/d*c* ^c^	calculated HHB	HBC naming
pHEAm	0.65	85	10.1	11.9	1.06	0.1696	46:54	HEAm 46:54
pGlcEAm	0.58	49	13.7	15.7	1.09	0.1556	53:47	Glc 53:47
pGalEAm	0.73	53	14.8	16.3	1.08	0.1451	54:46	Gal 54:46

a Conversion determined by 400 MHz ^1^H NMR spectroscopy in DMSO, relaxation delay = 5 s.

b Determined using GPC-MALLS in TRIS buffer (pH = 8.0) with a PL aquagel MIXED–OH column. A flow rate of 0.5 mL min^–1^ and a sample concentration of 20 mg mL^–1^.

c Hydrophilic PA d*n*/d*c* values determined using an offline refractometer at 25 °C.

Thiol–ene click coupling was utilized to prepare the block copolymers, as described in our previous publication, and detailed in the Supporting Information.^36^ Thiol–ene click coupling for the union of hydrophilic PAs and hydrophobic branched PLA was chosen as the method did not require postpolymerization modifications following PA deprotection, reduced the risk of thermal degradation, and offered the ability to avoid potential copper contamination common in copper catalyzed azide alkyne (CuAAC) click coupling reactions. An HHB target of 50:50 was chosen to produce micelles of similar compositions upon nanoprecipitation. Conversion was tracked by monitoring disappearance of vinyl peaks of PLA (Figure S9). No significant differences were detected during photocoupling reactions. HBCs were produced in 7 h with comparable yields and using similar purification procedures. A series of water washes to remove unreacted hydrophilic polymer followed by THF washes to remove unreacted hydrophobic polymer were employed as described in our previous report.^36^ Successful removal of unreacted homopolymers allowed HBCs to be isolated without any indication of degradation and successful incorporation of both blocks. ^1^H NMR spectra of starting blocks and coupled product are shown in Figure S10. The formation of stable nanostructures by nanoprecipitation supports the successful coupling of the blocks. HBC naming conventions are provided in Table 1.

### Nanoprecipitation, Nanostructure Morphology, and Aggregation Pathways

Nanoparticles were formed by the slow addition of the organic phase, HBC dissolved in THF, into DI water, following previously published procedures.^5,44^ The insolubility of PLA in hydrophilic environments drives the formation of micelles with hydrophobic core and hydrophilic shell. AFM and DLS, Figure 2 and Figure S12, respectively, were employed to evaluate the morphology and size of the nanostructures, and average diameters are compared in Table 2.

**Figure 2:**
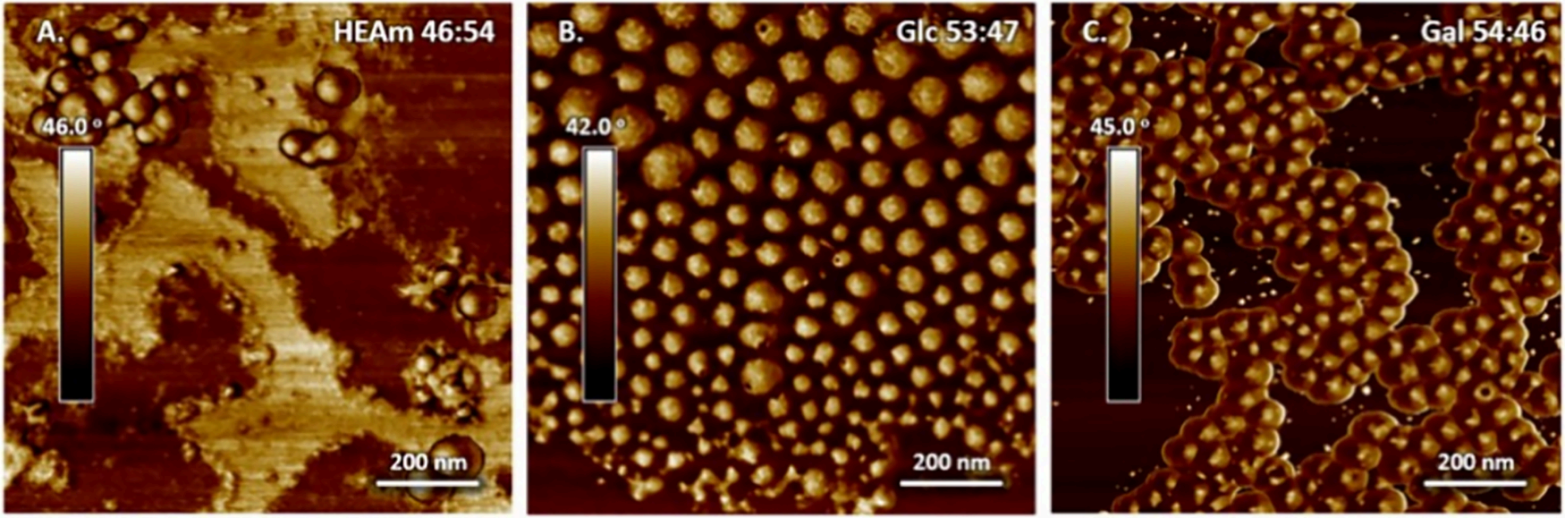
AFM phase images for (A) HEAm 46:54, (B) Glc 53:47, and (C) Gal 54:46 displaying spherical, solid, core–shell micelles of similar diameters formed via nanoprecipitation. Scale bars are 200 nm.

**Table 2:** Average Diameter of HBC Nanoparticles Formed in Water by Nanoprecipitation

sample	AFM (nm)^a^	DLS peak (nm)^b^	PDI^b^
HEAm 46:54	69 ± 12	140 ± 54	0.10 ± 0.011
Glc 53:47	64 ± 10	140 ± 62	0.14 ± 0.019
Gal 54:46	69 ± 2.4	120 ± 44	0.11 ± 0.0057

a Values represent mean ± one standard deviation of diameters measured via AFM

b Values determined using DLS software analysis.

Similar morphologies of comparable diameter are observed for the three systems, irrespective of the structure of the hydrophilic block. As expected for the approximately 50:50 HHB in each of the three HBCs, micellar self-assembled structures resulted from the self-assembly process, Figure 2. This indicates that the hydrophobic effect that drives nanoparticle formation is not overridden by secondary inter/intramolecular interactions within the hydrophilic block. It is also apparent that intramolecular interactions that promote exposure of the hydrophobic polyacrylamide backbone do not interfere with water solubility. Diameters measured by DLS are larger than those measured by AFM, as is often reported, attributed to the larger size of the particle plus the hydration shell measured in solution.^45,46^ Additionally, glycopolymers are prone to aggregation in solution resulting in larger particles, as reported widely by us and others, which may also contribute to the larger diameters measured in DLS.^36,37,36,37,47,48^

ATR-FTIR spectra shown in Figure S13 were acquired for dry powder saccharide containing HBCs in order to determine hydrogen bonding patterns (intermolecular vs intramolecular hydrogen bonds). The polymer backbone amide II band at 1518 cm^–1^ was used for normalization of the peak intensities.^37^ Spectra for saccharide-containing HBCs are similar except for the peaks between 1300 and 900 cm^–1^. Bristol et al. highlighted the impact of saccharide stereochemistry on altering aggregation processes and the effects of inter/intramolecular differences in IR vibrational spectra.^37^ It has been shown that carbohydrates with hydroxyls participating in intermolecular hydrogen bonding (glucose) typically have broader peaks at 1030 cm^–1^ in comparison to hydroxyls involved in intramolecular hydrogen bonding (galactose), which are narrower.^37,49,50^ These differences in hydrogen bonding interactions are consistent for homopolymers and HBCs.

### Thermal Analysis of HBCs

Thermal properties of dried, unassembled HBCs and homopolymers were determined via TGA (Figure S14) and DSC (Figure 3), and thermal transitions are summarized in Table 3. Thermal stability of HBCs, as well as of homopolymer precursors, was determined by TGA using dry powdered samples. Onset of degradation (*T*_d,5%_) is substantially lower for PLA (218 °C) than pHEAm (247 °C), with the values consistent with literature.^51,52^ Glycopolymers and glycopolymer HBCs display degradation onsets at significantly higher temperatures, ranging from 282 to 296 °C, whereas the HEAm 45:54 HBC has an intermediate *T*_d,5%_ of 271 °C. We attribute these differences to the increased chain rigidity upon incorporation of the bulky pendant saccharides, which leads to increased thermal stability.^53–55^

**Table 3:** Degradation (*T*_d,5%_), Glass Transition (*T*_g_), Crystalline (*T*_c_), and Melting (*T*_m_) Temperatures Determined by TGA and DSC

sample	*T*_d,5%_ (°C)	*T*_g_ (°C)	*T*_c_ (°C)	*T*_m_ (°C)
PLA	218	39		
pHEAm	247	108		
pGlcEAm	296	111		
pGalEAm	282	123		
HEAm 46:54	271	16		
Glc 53:47	286	27		
Gal 54:46	283	43	98	126, 137

**Figure 3:**
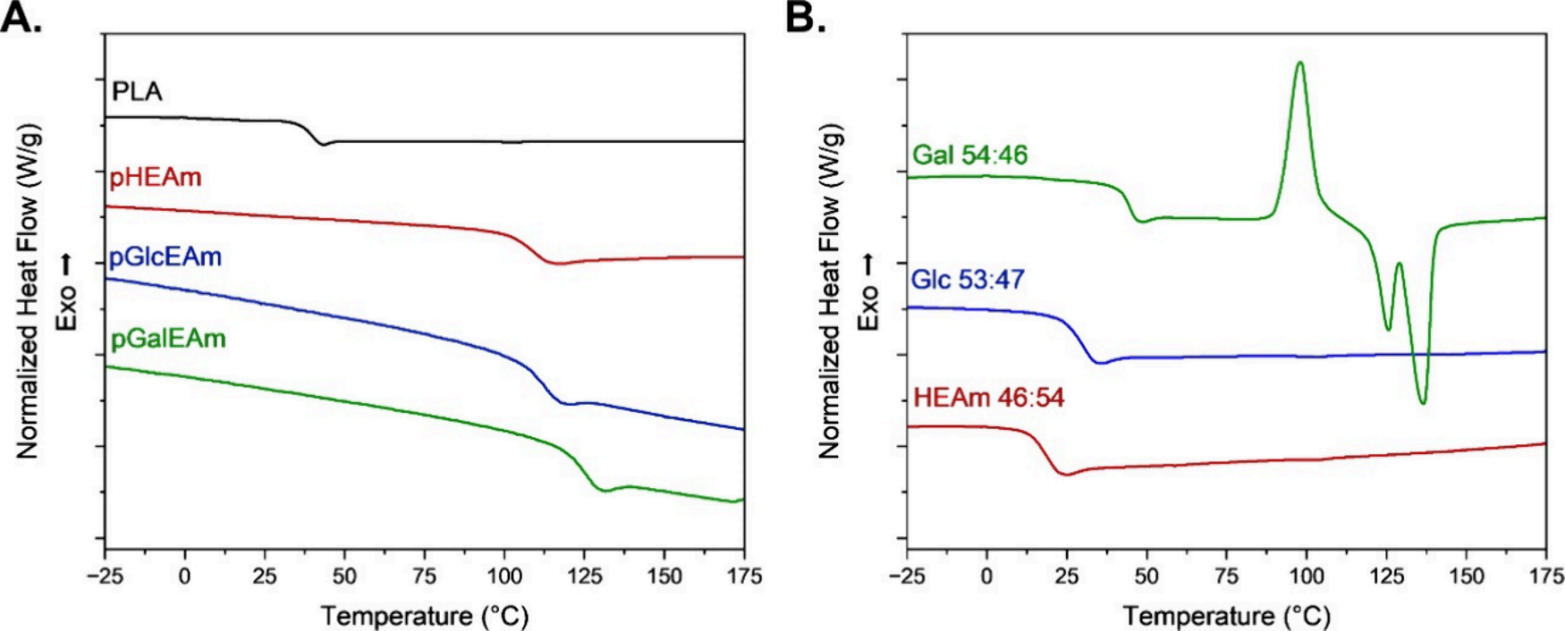
DSC curves, second heating, heating rate of 10 °C min^–1^: (A) starting material homopolymers and (B) amphiphilic HBCs ramped from −25 to 175 °C under nitrogen atmosphere displaying glass transition temperatures (*T*_g_) for all materials and a unique, multistage series of thermal transitions (*T*_c_, T_m,1_, and T_m,2_) for Gal 54:46.

DSC second heating curves for homopolymers are provided in Figure 3A and those for HBCs are shown in Figures 3B. PLA displays a *T*_g_ of 39 °C with no evidence of crystallization. This *T*_g_ value is consistent with literature reports for a branched PLA of this molecular weight. The absence of crystallization and melting peaks has been reported for highly branched PLAs and is attributed to steric bulk suppressing the crystallization process.^56^

Significantly higher *T*_g_ values, 108, 110, and 123 °C, are observed for the PA homopolymers pHEAm, pGlcEAm, and pGalEAm, respectively. The measured *T*_g_ for pHEAm is consistent with literature reports.^52^ The dramatically higher *T*_g_ for pGalEAm may be attributed to the propensity of the galactose pendant groups to participate in intramolecular bonding, reported previously by our group.^37^ This phenomenon may be associated with a decrease in the system’s free volume, as pendant galactose moieties favor intrachain associations that form densely packed structures and thus increase the overall glass transition temperature.^37,57^

DSC traces for the block copolymers are shown in Figure 3B. A single *T*_g_ is observed for HEAm 46:54 and Glc 53:47, in the temperature range of the PLA *T*_g_, with no evidence of crystallinity. The single *T*_g_ is attributed to phase mixing of the block copolymer after the second heating in DSC, as reported by Pustulka et al. for PLA block copolymers.^11^ In addition to *T*_g_, first-order transitions are observed in the Gal 54:46 HBC with an exothermic transition at 98.1 °C (*T*_c_) that is consistent with crystallization observed in linear PLA systems.^56^ A two-stage endothermic melting transition at 125.6 °C (*T*_m,1_) and 136.6 °C (*T*_m,2_) is also observed, similar to that for a thermoplastic starch (TPS)/PLA blend reported by Trinh et al.^58^ The initial (low-temperature) melting peak was attributed to a PLA/TPS entangled phase with greater chain mobility, the second to crystalline melting of PLA. Pustulka et al. observed crystalline transitions in the first heating cycle and only a single *T*_g_ on the second heating cycle.^11^ Similar behavior was observed for the glucose BCP, with a first order transition in the first heating cycle and a single *T*_g_ on the second cycle Figure S15A. In contrast, the galactose BCP showed one crystalline peak in the first heating cycle and additional crystalline peaks on the second heat Figure S15B.We hypothesize that the self-aggregating pGalEAm block copolymer promotes phase segregation and subsequent PLA crystallization by creating pGalEAm-rich domains that promote the molecular arrangement of PLA domains conducive to crystallization. Similar results have been reported in literature whereby increasing interactions promoted greater nucleation efficiency and accelerated crystallization.^59,60^

### Encapsulation Studies

Dye loading content and encapsulation efficiency for formulations with hydrophobic and hydrophilic dyes are shown in Table 4. Curcumin was chosen as a model hydrophobic drug due to its ease of detection via UV–vis methods.^6^ Curcumin is encapsulated upon self-assembly within the PLA hydrophobic core of the micelle. Curcumin alone is not soluble in water, and following overnight equilibration, precipitates completely from solution. There is no significant difference in DL or EE for the three HBCs, indicating that the structure of the hydrophilic block does not affect the encapsulation of curcumin. This is expected for the uptake of hydrophobic dyes in systems with equivalent HHB and constant hydrophobic blocks and is consistent with literature reports.^61–63^

**Table 4:** Loading and Encapsulation Efficiencies of Nanostructures with Curcumin and MO Dyes.^a^

	hydrophobic (curcumin)		hydrophilic (methyl orange)	
sample	DL (%)	EE (%)	DL (%)	EE (%)
HEAm 46:54	5.7 ± 0.72	10 ± 1	2.7 ± 0.21	1.8 ± 0.23
Glc 53:47	5.7 ± 0.72	10 ± 1	4.2 ± 0.21	2.0 ± 0.23
Gal 54:46	5.7 ± 0.72	10 ± 1	3.1 ± 0.21	2.0 ± 0.23

a Values represent mean ± one standard deviation.

Methyl orange (MO), a model negatively charged hydrophilic dye, was used for its ease of detection through UV–vis techniques and water solubility.^64^ As the micelles have a hydrophobic core, MO is absorbed solely through interactions with the hydrophilic corona. In general, DL% and EE% are lower for MO than for the hydrophobic dye (*p* < 0.05). HEAm shows lower DL% than both saccharide copolymers (*p* < 0.05), and Glc shows greater DL % than Gal (*p* < 0.08) The lower DL for HEAm may be explained by its overall lower hydrophilicity and hydroxyl content than the glycopolymers.^6,64–66^ The slightly higher DL for Glc 53:47 than Gal 54:46 may be related to the Glc polymer’s greater propensity for intermolecular hydrogen bonding, which promotes more interaction with the guest molecule.^6,37,63^

### Lectin Binding Properties of Self-Assembled Nanostructures

To understand the capabilities of these systems for *in vivo* targeting, lectin binding profiles were investigated using QCM-D; this data is presented in Figure 4. Peanut agglutinin (PNA) was used in this study as a model lectin because it is known to have selective affinity for β-galactose.^34^ By first grafting the PNA lectins to the modified gold QCM-D sensors, an initial drop in frequency, indicating mass addition, was observed. Once a stable baseline was achieved, a series of washes were conducted to confirm the successful addition of PNA lectin and to protect the unreacted NHS present on the sensor surface.

**Figure 4:**
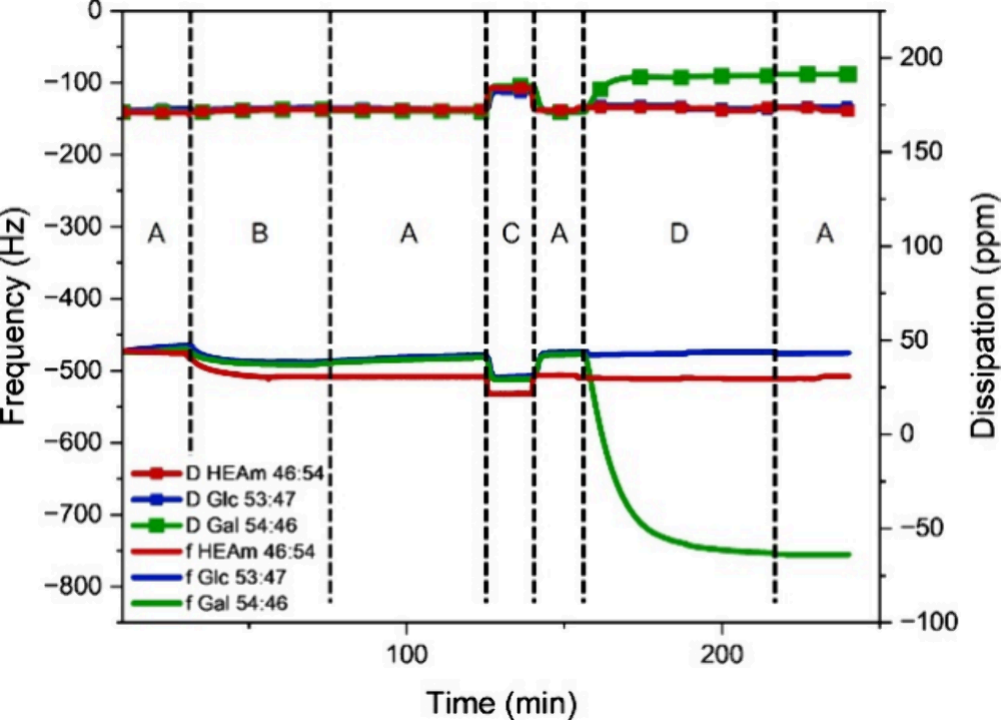
QCM-D third harmonic plot of multilayer deposition cycles showing changes of frequency, Δ*f*, and dissipation, Δ*D*, over time. Flow regimes of surface modification are (A) HBS buffer, (B) 0.5 mg mL^–1^ PNA lectin in buffer, (C) 1 M ethanolamine HCl, and (D) 0.25 mg mL^–1^ nanostructures in HBS buffer. Legend denoted that solid lines are frequency (*f*), left axis, and lines with squares are dissipation (*D*), right axis. As expected, only the galactose-containing HBC shows significant binding to PNA, observed as a large decrease in frequency.

Upon introducing solutions of the HBC nanostructures, a significant decrease in frequency, indicating mass adsorption, and a small increase in dissipation were observed exclusively for the Gal 54:46, shown in Figure 4. This specific binding response suggests successful surface orientation and preservation of the β-d-galactose moieties throughout synthesis and characterization. This enhanced binding efficiency may be due to the multivalent nature of HBCs.^29,30^

### Nanostructure Cytotoxicity

To assess the cytotoxicity of the newly formed HBC nanostructures, HEK293 cells were exposed to these structures for 24 h, after which the release of lactate dehydrogenase (LDH) was quantified. For comparative purposes, PEG, known for its biocompatibility and hydrophilic properties, served as the control. The results depicted in Figure 5 indicate that all tested formulations exhibited minimal cytotoxicity, with values remaining below 10% across various concentrations, and no significant variations noted among the treatments. Fluorescent microscopy images, obtained after conducting live/dead staining, shown in Figure 5, further support these findings by showing minimal cell mortality when compared to control samples. This highlights the potential use of HBCs as safe and noncytotoxic carriers for the delivery of drugs and dyes.

**Figure 5:**
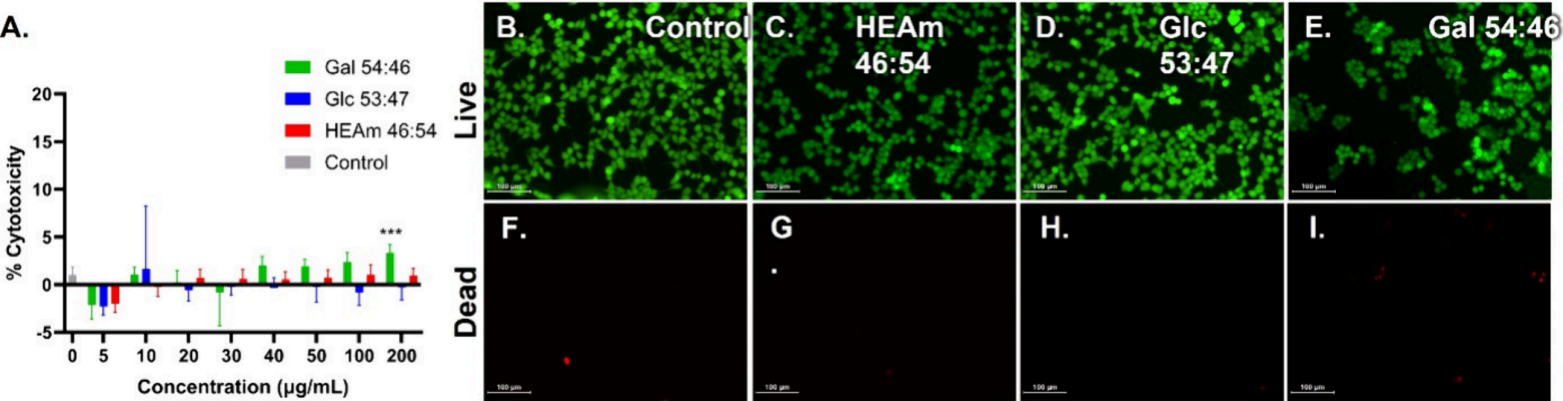
(A) Nanoparticle cytotoxicity after 24 h of exposure in HEK 293 cells determined using an LDH quantification assay. No significant differences are observed between PEG and self-assembled glyco-nanoparticles at all concentrations tested. Data bars represent the mean ± one standard deviation (s.d.) (*n* = 6 per treatment). Analysis of variance (ANOVA) with a Tukey post hoc test was used to assess significance (*p < 0.05), where significance was reported for increases in toxicity from the control. (B–I) Fluorescence imaging of cells treated with 100 μg/mL of sample for 24 h. (B–E) represent live cells stained with calcein-AM. (F–I) represent dead cells stained with BOBO-3. Images (B) and (F) are controls, and (C) and (G) were incubated with HEAm 46:54, (D) and (H) with Glc 53:47, and (E) and (I) with Gal 54:46 nanoparticles. Scale bars are 100 μm.

## Conclusions

HBCs of branched PLA coupled to hydrophilic acrylamide blocks with varying pendant groups displayed low cytotoxicity and similar self-assembly behaviors but exhibited differences in thermal properties, dye uptake, and lectin-binding interactions. At an approximate HHB of 50:50, PLA copolymers with hydroxyethyl, β-d-glucose, and β-d-galactose polyacrylamide blocks produced spherical core–shell micelles of comparable diameter. Glycopolyacrylamide blocks possessed higher *T*_g_s and degradation temperatures than the pHEAm block. Remarkably, the galactose pendant block promoted crystallization of the PLA phase in the melt state, in contrast to the homopolymer branched PLA and other block copolymers that showed no crystallinity. We attribute this behavior to the known preferential intramolecular association of pGalEAm, which may have induced phase separation and facilitated PLA crystallization. Small differences observed in hydrophilic dye uptake may be related to galactose intramolecular associations, with no differences in hydrophobic dye uptake noted. Preferential binding of Gal 54:46 to PNA demonstrates the potential for cell selectivity in therapeutic delivery, such as galectins found in malignant cells in the stomach and liver. These studies provide an enhanced understanding of the effects of saccharide structure and stereochemistry on glycopolymer HBC properties, with implications for the optimization of cargo loading, nanostructure stability, and delivery. The combined findings demonstrate the high potential of glycopolymer HBCs for the design of noncytotoxic targeted drug delivery vehicles.

## Supplementary Material



## Notes

Synthesis and NMR characterization of HEAm, acetylated glycomonomers (AcGlcEAm and AcGalEAm), and PA homopolymers (pHEAm, pGlcEAm, and pGalEAm); polymerization kinetics plots; UV–vis absorption spectra of PA homopolymers; synthesis and NMR characterization of PLA; synthesis of HBCs; characterization of homopolymer and block copolymer structures by NMR; dye absorbance spectra; DLS of nanoparticles; ATR-FTIR spectra of glyco-HBCs; TGA thermograms of homopolymers and block copolymers; DSC heating and cooling curves of block copolymers; gold QCM-D sensor grafting process; cell viability and live/dead imaging methods (PDF)

## References

[1] Sadeghi A., PourEskandar S., Askari E., Akbari M. (2023). Polymeric Nanoparticles
and Nanogels: How Do They Interact with Proteins?. Gels.

[2] Kalita H., Patowary M. (2023). Biocompatible Polymer
Nano-Constructs: A Potent Platform
for Cancer Theranostics. Technol. Cancer Res.
Treat..

[3] Kamaly N., Yameen B., Wu J., Farokhzad O. C. (2016). Degradable
Controlled-Release Polymers and Polymeric Nanoparticles: Mechanisms
of Controlling Drug Release. Chem. Rev..

[4] Chandrasiri I., Abebe D. G., Gupta S., Williams J. S. D., Rieger W. D., Simms B. L., Yaddehige M. L., Noh Y., Payne M. E., Fortenberry A. W., Smith A. E., Ilavsky J., Grayson S. M., Schneider G. J., Watkins D. L. (2019). Synthesis and characterization of
polylactide-PAMAM “Janus-type” linear-dendritic hybrids. J. Polym. Sci., Part A: Polym. Chem..

[5] Chandrasiri I., Abebe D. G., Loku
Yaddehige M., Williams J. S. D., Zia M. F., Dorris A., Barker A., Simms B. L., Parker A., Vinjamuri B. P., Le N., Gayton J. N., Chougule M. B., Hammer N. I., Flynt A., Delcamp J. H., Watkins D. L. (2020). Self-Assembling
PCL–PAMAM Linear Dendritic Block Copolymers (LDBCs) for Bioimaging
and Phototherapeutic Applications. ACS Appl.
Bio Mater..

[6] Chandrasiri I., Loku Yaddehige M., Li B., Sun Y., Meador W. E., Dorris A., Farid Zia M., Hammer N. I., Flynt A., Delcamp J. H., Davis E., Lippert A., Watkins D. L. (2022). Cross-linking
Poly­(caprolactone)–Polyamidoamine Linear Dendritic Block Copolymers
for Theranostic Nanomedicine. ACS Appl. Polym.
Mater..

[7] Yaddehige M. L., Chandrasiri I., Barker A., Kotha A. K., Dal Williams J. S., Simms B., Kucheryavy P., Abebe D. G., Chougule M. B., Watkins D. L. (2020). Structural and Surface Properties of Polyamidoamine
(PAMAM) – Fatty Acid-based Nanoaggregates Derived from Self-assembling
Janus Dendrimers. ChemNanoMat.

[8] Chauhan A. S. (2018). Dendrimers
for Drug Delivery. Molecules.

[9] Gouveia M. G., Wesseler J. P., Ramaekers J., Weder C., Scholten P. B. V., Bruns N. (2023). Polymersome-based protein
drug delivery - quo vadis?. Chem. Soc. Rev..

[10] Johnson B.
K., Prud’homme R. K. (2003). Mechanism
for Rapid Self-Assembly of Block Copolymer
Nanoparticles. Phys. Rev. Lett..

[11] Pustulka K. M., Wohl A. R., Lee H. S., Michel A. R., Han J., Hoye T. R., McCormick A. V., Panyam J., Macosko C. W. (2013). Flash Nanoprecipitation:
Particle Structure and Stability. Mol. Pharm..

[12] Mann J., Mayer J. K., Garnweitner G., Schilde C. (2023). Influence of Process
Parameters on the Kinetics of the Micelle-to-Vesicle Transition and
Ripening of Polystyrene-Block-Polyacrylic Acid. Polymers.

[13] Bovone G., Cousin L., Steiner F., Tibbitt M. W. (2022). Solvent Controls
Nanoparticle Size during Nanoprecipitation by Limiting Block Copolymer
Assembly. Macromolecules.

[14] de
Oliveira F. A., C. S. Batista C., J. C. Albuquerque L., Černoch P., Steinhart M., Sincari V., Jager A., Jager E., Giacomelli F. C. (2023). Tuning the morphology of block copolymer-based
pH-triggered nanoplatforms as driven by changes in molecular weight
and protocol of manufacturing. J. Colloid Interface
Sci..

[15] Ford J., Chambon P., North J., Hatton F. L., Giardiello M., Owen A., Rannard S. P. (2015). Multiple
and Co-Nanoprecipitation
Studies of Branched Hydrophobic Copolymers and A–B Amphiphilic
Block Copolymers, Allowing Rapid Formation of Sterically Stabilized
Nanoparticles in Aqueous Media. Macromolecules.

[16] González-Pastor R., Lancelot A., Morcuende-Ventura V., San Anselmo M., Sierra T., Serrano J. L., Martin-Duque P. (2021). Combination
Chemotherapy with Cisplatin and Chloroquine: Effect of Encapsulation
in Micelles Formed by Self-Assembling Hybrid Dendritic-Linear-Dendritic
Block Copolymers. Int. J. Mol. Sci..

[17] Gupta A., Costa A. P., Xu X., Lee S. L., Cruz C. N., Bao Q., Burgess D. J. (2020). Formulation and characterization of curcumin loaded
polymeric micelles produced via continuous processing. Int. J. Pharm..

[18] Hu C., Chen Z., Wu S., Han Y., Wang H., Sun H., Kong D., Leng X., Wang C., Zhang L., Zhu D. (2017). Micelle or polymersome formation by PCL-PEG-PCL copolymers as drug
delivery systems. Chin. Chem. Lett..

[19] Moros M., Hernáez B., Garet E., Dias J. T., Sáez B., Grazú V., González-Fernández Á., Alonso C., de la Fuente J. M. (2012). Monosaccharides versus PEGFunctionalized
NPs: Influence in the Cellular Uptake. ACS Nano.

[20] Neun B. W., Barenholz Y., Szebeni J., Dobrovolskaia M. A. (2018). Understanding
the Role of Anti-PEG Antibodies in the Complement Activation by Doxil
in Vitro. Molecules.

[21] Ahmed M., Lai B. F., Kizhakkedathu J. N., Narain R. (2012). Hyperbranched glycopolymers
for blood biocompatibility. Bioconjugate Chem..

[22] Kang B., Opatz T., Landfester K., Wurm F. R. (2015). Carbohydrate nanocarriers
in biomedical applications: functionalization and construction. Chem. Soc. Rev..

[23] Bernardes G. J., Kikkeri R., Maglinao M., Laurino P., Collot M., Hong S. Y., Lepenies B., Seeberger P. H. (2010). Design,
synthesis and biological evaluation of carbohydrate-functionalized
cyclodextrins and liposomes for hepatocyte-specific targeting. Org. Biomol. Chem..

[24] Buzzacchera I., Xiao Q., Han H., Rahimi K., Li S., Kostina N. Y., Toebes B. J., Wilner S. E., Möller M., Rodriguez-Emmenegger C., Baumgart T., Wilson D. A., Wilson C. J., Klein M. L., Percec V. (2019). Screening Libraries of Amphiphilic
Janus Dendrimers Based on Natural Phenolic Acids to Discover Monodisperse
Unilamellar Dendrimersomes. Biomacromolecules.

[25] Delbianco M., Bharate P., Varela-Aramburu S., Seeberger P. H. (2016). Carbohydrates
in Supramolecular Chemistry. Chem. Rev..

[26] Liu Z., Jiao Y., Wang Y., Zhou C., Zhang Z. (2008). Polysaccharides-based
nanoparticles as drug delivery systems. Adv.
Drug Delivery Rev..

[27] Ladmiral V., Semsarilar M., Canton I., Armes S. P. (2013). Polymerization-induced
self-assembly of galactose-functionalized biocompatible diblock copolymers
for intracellular delivery. J. Am. Chem. Soc..

[28] Yan X., Chai L., Fleury E., Ganachaud F., Bernard J. (2021). ‘Sweet as a Nut’: Production
and use
of nanocapsules made of glycopolymer or polysaccharide shell. Prog. Polym. Sci..

[29] Rydell G. E., Dahlin A. B., Hook F., Larson G. (2009). QCM-D studies of human
norovirus VLPs binding to glycosphingolipids in supported lipid bilayers
reveal strain-specific characteristics. Glycobiology.

[30] Pröhl M., Seupel S., Sungur P., Höppener S., Gottschaldt M., Brendel J. C., Schubert U. S. (2017). The influence of
the grafting density of glycopolymers on the lectin binding affinity
of block copolymer micelles. Polymer.

[31] Raposo C. D., Canelas A. B., Barros M. T. (2021). Human Lectins,
Their Carbohydrate
Affinities and Where to Find Them. Biomolecules.

[32] Sun P., Lin M., Zhao Y., Chen G., Jiang M. (2015). Stereoisomerism
effect
on sugar-lectin binding of self-assembled glyco-nanoparticles of linear
and brush copolymers. Colloids Surf., B.

[33] Das P. K., Dean D. N., Fogel A. L., Liu F., Abel B. A., McCormick C. L., Kharlampieva E., Rangachari V., Morgan S. E. (2017). Aqueous RAFT Synthesis of Glycopolymers
for Determination
of Saccharide Structure and Concentration Effects on Amyloid beta
Aggregation. Biomacromolecules.

[34] Gou Y., Slavin S., Geng J., Voorhaar L., Haddleton D. M., Becer C. R. (2012). Controlled Alternate Layer-by-Layer Assembly of Lectins
and Glycopolymers Using QCM-D. ACS Macro Lett..

[35] Gou Y., Richards S. J., Haddleton D. M., Gibson M. I. (2012). Investigation of
glycopolymer–lectin interactions using QCM-d: comparison of
surface binding with inhibitory activity. Polym.
Chem..

[36] Green K. A., Kulkarni A. S., Jankoski P. E., Newton T. B., Derbigny B., Clemons T. C., Watkins D. L., Morgan S. E. (2024). Biocompatible glycopolymer-PLA
amphiphilic hybrid block copolymers with unique self-assembly, uptake,
and degradation properties. Biomacromolecules.

[37] Bristol A. N., Saha J., George H. E., Das P. K., Kemp L. K., Jarrett W. L., Rangachari V., Morgan S. E. (2020). Effects of Stereochemistry
and Hydrogen Bonding on Glycopolymer-Amyloid-beta Interactions. Biomacromolecules.

[38] Stockmal K. A., Downs L. P., Davis A. N., Kemp L. K., Karim S., Morgan S. E. (2022). Cationic Glycopolyelectrolytes
for RNA Interference
in Tick Cells. Biomacromolecules.

[39] Saha J., Dean D. N., Dhakal S., Stockmal K. A., Morgan S. E., Dillon K. D., Adamo M. F., Levites Y., Rangachari V. (2021). Biophysical
characteristics of lipid-induced Aβ oligomers correlate to distinctive
phenotypes in transgenic mice. FASEB J..

[40] Xi W., Dean D. N., Stockmal K. A., Morgan S. E., Hansmann U. H. E., Rangachari V. (2019). Large fatty
acid-derived Aβ42 oligomers form
ring-like assemblies. J. Chem. Phys..

[41] Ciuca M. D., Racovita R. C. (2023). Curcumin: Overview
of Extraction Methods, Health Benefits,
and Encapsulation and Delivery Using Microemulsions and Nanoemulsions. Int. J. Mol. Sci..

[42] Uskoković V. (2023). Supplementation
of Polymeric Reservoirs with Redox-Responsive Metallic Nanoparticles
as a New Concept for the Smart Delivery of Insulin in Diabetes. Materials.

[43] Voinova M. V., Rodahl M., Jonson M., Kasemo B. (1999). Viscoelastic Acoustic
Response of Layered Polymer Films at Fluid-Solid Interfaces: Continuum
Mechanics Approach. Phys. Scr..

[44] Adharis A., Ketelaar T., Komarudin A. G., Loos K. (2019). Synthesis and Self-Assembly
of Double-Hydrophilic and Amphiphilic Block Glycopolymers. Biomacromolecules.

[45] Zhang C., Han D. J., Lee D. H., Lee H. H., Lee J.-K., Lee B. Y. (2024). Comparison of atomic
force microscopy and dynamic light
scattering on the size characterization of surfactant-modified WC/Co
nanoparticles dispersed in aqueous media. Colloids
Surf., A.

[46] MacCuspie R. I., Rogers K., Patra M., Suo Z., Allen A. J., Martin M. N., Hackley V. A. (2011). Challenges for physical characterization
of silver nanoparticles under pristine and environmentally relevant
conditions. J. Environ. Monit..

[47] Zheng Z., Wang B., Chen J., Wang Y., Miao Z., Shang C., Zhang Q. (2021). Facile synthesis
of Antibacterial,
Biocompatible, quaternized Poly­(ionic liquid)­s with pendant saccharides. Eur. Polym. J..

[48] Houga C., Giermanska J., Lecommandoux S., Borsali R., Taton D., Gnanou Y., Le Meins J. (2009). Micelles and Polymersomes Obtained
by Self-Assembly of Dextran and Polystyrene Based Block Copolymers. Biomacromolecules.

[49] Hu J., Chen C., Lu Z., Ma J., Cheng K., Lv J., Zeng K., Yang G. (2022). The role of intramolecular and intermolecular
hydrogen bonding effect for adenine-containing polyimide films. High Perform. Polym..

[50] Vasko P., Blackwell J., Koenig J. (1971). Infrared and raman spectroscopy of
carbohydrates: Part I: Identification of OH and CH-related vibrational
modes for D-glucose, maltose, cellobiose, and dextran by deuterium-substitution
methods. Carbohyd. Res..

[51] Zhao X., Li J., Liu J., Zhou W., Peng S. (2021). Recent progress of
preparation of branched poly­(lactic acid) and its application in the
modification of polylactic acid materials. Int.
J. Biol. Macromol..

[52] Narumi A., Chen Y., Sone M., Fuchise K., Sakai R., Satoh T., Duan Q., Kawaguchi S., Kakuchi T. (2009). Poly­(N-hydroxyethylacrylamide) Prepared
by Atom Transfer
Radical Polymerization as a Nonionic, Water-Soluble, and Hydrolysis-Resistant
Polymer and/or Segment of Block Copolymer with a Well-Defined Molecular
Weight. Macromol. Chem. Phys..

[53] Saltan F., Akat H. (2013). Synthesis and thermal degradation kinetics of D (+) galactose containing
polymers. Polímeros.

[54] Cerrada M. L., Sánchez-Chaves M., Ruiz C., Fernández-García M. (2008). Glycopolymers
resultant from ethylene–vinyl alcohol copolymers: Degradation
and rheological behavior in bulk. Eur. Polym.
J..

[55] Gregory G. L., Lopez-Vidal E. M., Buchard A. (2017). Polymers from sugars: cyclic monomer
synthesis, ring-opening polymerisation, material properties and applications. Chem. Commun..

[56] Shen W., Zhang G., Ge X., Li Y., Fan G. (2018). Effect on
electrospun fibres by synthesis of high branching polylactic acid. R. Soc. Open Sci..

[57] Origlia M. L., Call T. G., Woolley E. M. (2000). Apparent
molar volumes and apparent
molar heat capacities of aqueous d-glucose and d-galactose at temperatures
from 278.15 to 393.15 K and at the pressure 0.35 MPa. J. Chem. Thermodynamics.

[58] Trinh B. M., Tadele D. T., Mekonnen T. H. (2022). Robust
and high barrier thermoplastic
starch – PLA blend films using starch-graft-poly­(lactic acid)
as a compatibilizer. Mater. Adv..

[59] Bao J., Chang X., Shan G., Bao Y., Pan P. (2016). Synthesis
of end-functionalized hydrogen-bonding poly­(lactic acid)­s and preferential
stereocomplex crystallization of their enantiomeric blends. Polym. Chem..

[60] Yu M.-M., Yang W.-J., Niu D.-Y., Cai X.-X., Weng Y.-X., Dong W.-F., Chen M.-Q., Xu P.-W., Wang Y., Chu H., Ma P.-M. (2021). Enhancing
the Crystallization Performance of Poly­(L-lactide)
by Intramolecular Hybridizing with Tunable Self-assembly-type Oxalamide
Segments. Chin. J. Polym. Sci..

[61] Ahmed S. E., Fletcher N. L., Prior A. R., Huda P., Bell C. A., Thurecht K. J. (2022). Development of targeted
micelles and polymersomes prepared
from degradable RAFT-based diblock copolymers and their potential
role as nanocarriers for chemotherapeutics. Polym. Chem..

[62] Alibolandi M., Ramezani M., Abnous K., Sadeghi F., Hadizadeh F. (2015). Comparative
evaluation of polymersome versus micelle structures as vehicles for
the controlled release of drugs. J. Nanopart.
Res..

[63] Buwalda S., Al Samad A., El Jundi A., Bethry A., Bakkour Y., Coudane J., Nottelet B. (2018). Stabilization
of poly­(ethylene glycol)-poly­(epsilon-caprolactone)
star block copolymer micelles via aromatic groups for improved drug
delivery properties. J. Colloid Interface Sci..

[64] Goel S., Jacob J. (2020). D-galactose-based organogelator
for phase-selective solvent removal
and sequestration of cationic dyes. React. Funct.
Polym..

[65] Wang H., Calubaquib E. L., Bhadran A., Ma Z., Miller J. T., Zhang A., Biewer M. C., Stefan M. C. (2023). Self-assembly behavior
of thermoresponsive difunctionalized γ-amide polycaprolactone
amphiphilic diblock copolymers. Polym. Chem..

[66] Cai K., He X., Song Z., Yin Q., Zhang Y., Uckun F. M., Jiang C., Cheng J. (2015). Dimeric drug
polymeric nanoparticles
with exceptionally high drug loading and quantitative loading efficiency. J. Am. Chem. Soc..

