# Supplementary material for: Influence of the Saccharide Structure on Cargo Loading, Thermal Properties, and Lectin Binding of Amphiphilic Glycopolymer-Polylactic Acid Block Copolymer Nanoparticles
Source: Bioconjug Chem. 2025 Jul 17;36(8):1733–43. doi: 10.1021/acs.bioconjchem.5c00217 (PMC12371692; doi:10.1021/acs.bioconjchem.5c00217)
Supplement: Supplementary file 1 [file bc5c00217_si_001.pdf]

Supplemental Information

Influence of saccharide structure on cargo loading, thermal properties, and lectin binding of amphiphilic glycopolymer-PLA block copolymer nanoparticles

Kevin A. Green[1], Anuja S. Kulkarni[2,3], Penelope E. Jankoski[1], Rachel M. Worden[1,4], Bayleigh M. Loving,[1] Blaine Derbigny[2,3], Tristan D. Clemons[1], Davita L. Watkins[2,3], Sarah E. Morgan[1*]*

[1]School of Polymer Science and Engineering, The University of Southern Mississippi, Hattiesburg, Mississippi 39406, United States

[2]Department of Chemistry & Biochemistry, The Ohio State University, Columbus, OH 43210, United States

[3]William G. Lowrie Department of Chemical and Biomolecular Engineering, The Ohio State University, 151 W Woodruff Ave., Columbus, OH 43210, USA

[4]The Georgia Institute of Technology, Department of Chemistry and Biochemistry, Atlanta, GA, 30318

*Corresponding authors: **watkins.891@osu.edu**, **sarah.morgan@usm.edu**

1.1 Glycomonomer Synthesis. The acetyl protected glucose and galactose pendant acrylamide monomer, 2′-acrylamidoethyl-2,3,4,6-tetra-O-acetyl-β-D-glucopyranoside (AcGlcEAm) and 2′-acrylamidoethyl-2,3,4,6-tetra-O-acetyl-β-D-galactopyranoside (AcGalEAm), were synthesized following previous literature procedures (Scheme S.1).[1-3] In short, N-hydroxyethyl acrylamide (21.00 g, 182.4 mmol) in excess was added with acetobromo-α-D-glucose or α-D-galactose (15.00 g, 36.5 mmol) in distilled DCM (400 mL) to a 1000 mL, 3-neck round-bottom flask. Dry molecular sieves (20.00 g, 4 Å size) were added to the reaction mixture to ensure that the reaction was completely dry. The reaction mixture was submerged in an ice bath and allowed to mix for 30 minutes using a mechanical stirrer. At this point, a metal catalyst, silver trifluoromethanesulfonate (14.00 g, 52.2 mmol), was added, and the reaction was allowed to proceed for 48 hours in the dark. The reaction mixture was then filtered, and the crude solution was washed with 1 M HCl (3 x 100 mL), washed with deionized (DI) water (3 x 100 mL), dried over sodium sulfate, and filtered once

more. The collected liquid was concentrated through rotary evaporation and purified through column chromatography (SiO_2, 10:1 EtOAc:Hexanes). Fractions collected after purification were then completely dried through rotary evaporation to afford a white crystalline solid (5.49 g, 33.8%). ^{1}H NMR (400 MHz, CDCl3) δ [ppm] 1.97, 2.03, 2.14 (s, s, s, 12H-13, 14, 15, 16), 3.57 (m, 2H-5), 3.70 (m, 1H-9), 3.91 (m, 2H-4), 4.14 (m, 2H-11, 12), 4.48 (m, 1H-6), 5.02 (d of d, 1H-10), 5.16 (m, 1H-7), 5.38 (t, 1H-8), 5.66 (d of d, 1H-1), 6.05 (m, 1H-2), 6.31 (m, 1H-3).

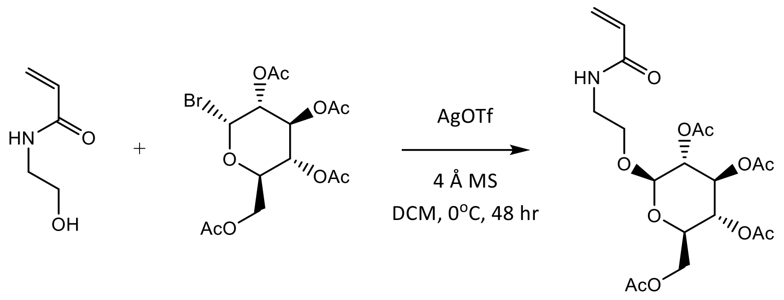

Scheme S 1. Synthesis of acetal protected glycomonomers with example AcGlcEAm.

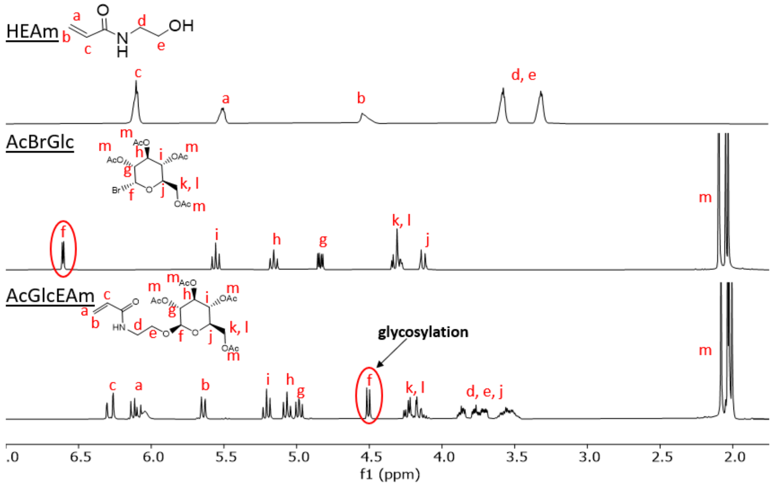

Figure S1. ^{1}H NMR spectra comparison of HEAm, AcBrGlc, and AcGlcEAm in $CDCl_3$.

1.2 RAFT Polymerization of Hydrophilic Polymers. HEAm, Glucose, and Galactose functionalized polymers (pHEAm, pGlcEAm, and pGalEAm) were synthesized similar to previous literature procedures (Scheme S.2).[3] Polymers with a target degree of polymerization (DP) of 120 or 55 (molecular weight = 14,000 g/mol) were synthesized by RAFT polymerization. The monomer (HEAm) or acetyl-protected glycomonomer (AcGlcEAm or AcGalEAm), chain

transfer agent (CEP), thermal initiator (V-501), and internal standard (trimesic acid) were dissolved in anhydrous DMSO in a 25-mL round-bottom flask equipped with a magnetic stirring bar and degassed with ultrapure N_2 for 45 minutes while stirring. All polymerizations were carried out at 0.4 M initial monomer concentration. The $[M]_0$:$[CTA]_0$ varied depending on the target molecular weight for each reaction, while the $[CTA]_0$:$[I]_0$ was maintained at 5:1. Reactions were carried out to achieve a final monomer conversion of 60%. To determine reaction times needed to achieve desired molecular weights, kinetic studies were performed where the disappearance of the vinyl monomer peaks (6.0 ppm, 2H and 5.5 ppm, 1H) was monitored by ^{1}H NMR spectroscopy (Fig. S.2) and compared to the carboxylic acid peak (8.6 ppm, 3H) of trimesic acid at different reaction times. After the target molecular weights were achieved, the reaction was quenched by submerging the flask into LN_2 and precipitating the reaction mixture into DI water. The liquid was decanted leaving behind a yellow solid (pAcGlcEAm). In order to remove both the acetyl-protecting groups and the trithiol end group, residual from CEP, the polymer was dissolved in methanol (25 mL) and sodium methoxide was added in excess while stirring for 1 hour. The deprotected polymer precipitated from the solution, this mixture was centrifuged, and the polymer was then isolated by decanting the remaining liquid. The solid polymer was redissolved in DI water, dialyzed using Spectrum™ Labs Spectra/Por™ 3.5 kDa MWCO standard RC tubing for 3 days in DI water, and lyophilized to isolate the pGlcEAm as a white powder (72% yield). ^{1}H NMR (600 MHz, D_2O) δ [ppm] 3.49−4.11 (m, 10H4, 5, 7, 8, 9, 10, 11, 12), 4.39 (d, 1H-6), 5.75 (d of d, 1H-1), 6.19 (m, 1H-3, 1H-2).

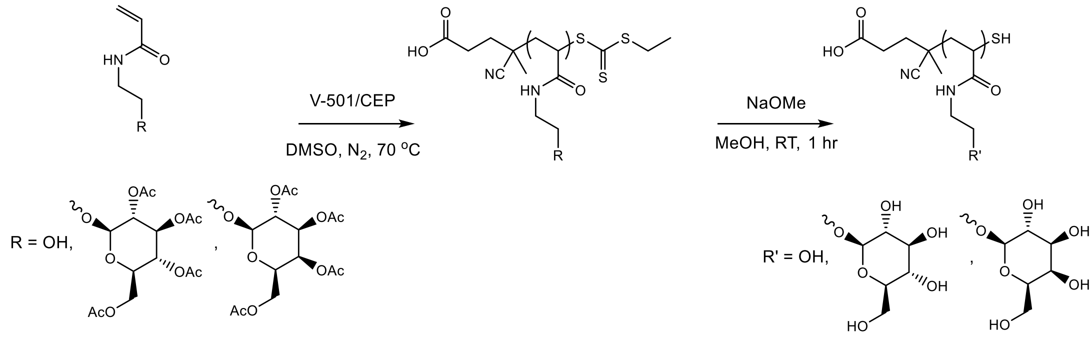

Scheme S2. Synthesis of HEAm, glucose, or galactose-functionalized glycopolymers via RAFT polymerization and base-catalyzed deprotection.

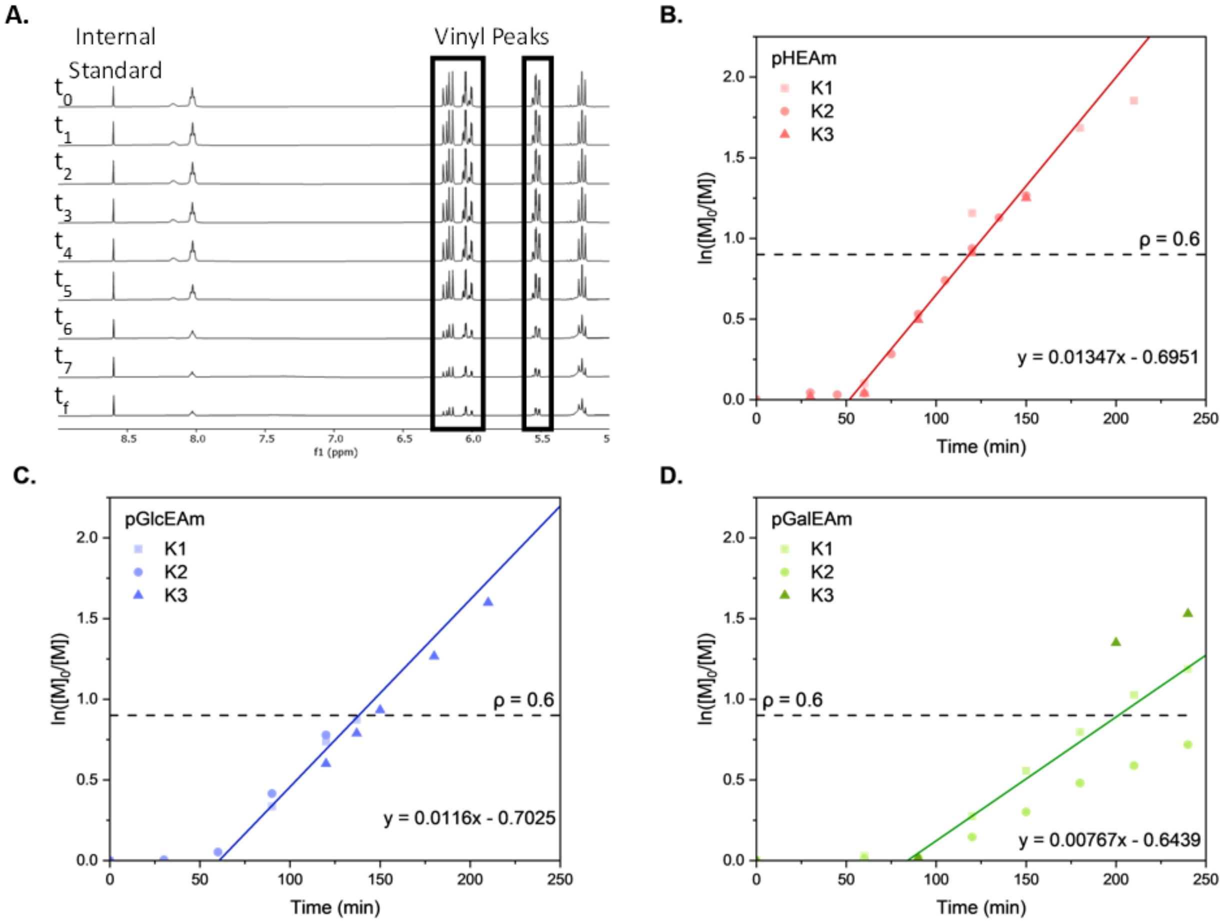

Figure S2. A) Example ^{1}H NMR spectra of the RAFT polymerization of glucose-functionalized glycopolymers tracking conversion over time in DMSO-d6. Plots of ln([M]o/[M]) vs. time for RAFT polymerization of B) pHEAm, C) pAcGlcEAm, and D) pAcGalEAm with CEP and V-501 at 70°C in DMSO. The dotted line represents a monomer conversion (ρ) of 60%. The solid line represents averaged kinetics for target polymerization conditions.

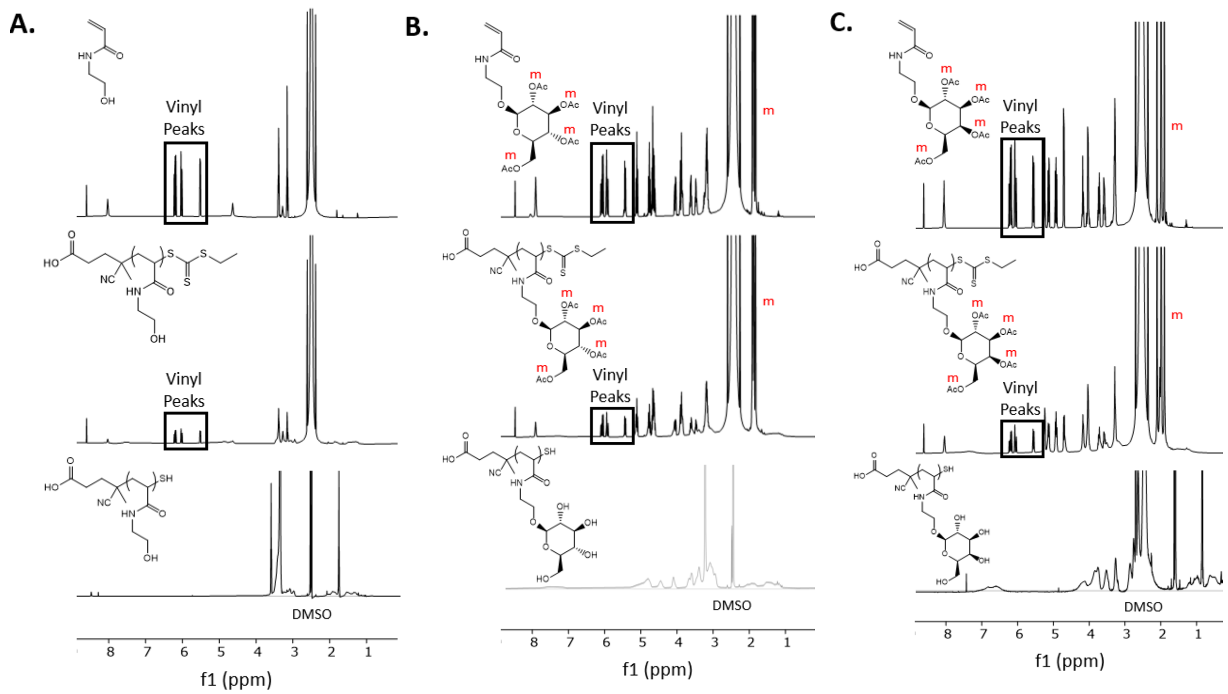

Figure S3. ^{1}H NMR spectra comparison for A) HEAm, pHEAm – trithiol, and pHEAm – SH, B) AcGlcEAm, pAcGlcEAm, and pGlcEAm and C) AcGalEAm, pAcGalEAm, and pGalEAm in DMSO-d_6.

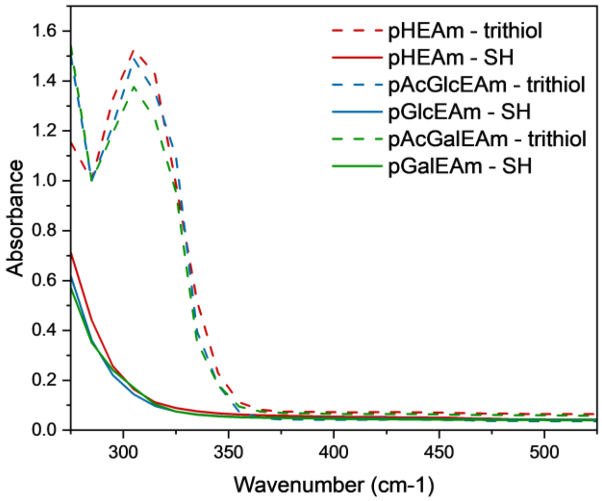

Figure S4. UV-Vis absorption spectra of protected and deprotected homopolymers in DMSO. The decrease in absorbance at 308 nm indicates cleavage of trithiocarbonate chain ends following base-catalyzed deprotection.

1.3 PLA Synthesis

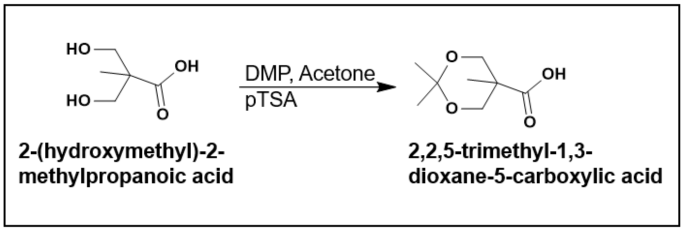

Scheme S3. Synthesis of 2,2,5-trimethyl-1,3-dioxane-5-carboxylic acid.

This product was synthesized following a previously reported procedure.[4] Briefly, 2-(hydroxymethyl)-2-methylpropanoic acid (Bis-MPA) (10 g, 75 mmol) was stirred in 50 mL of acetone, to it 2,2-dimethoxypropane (DMP) (11.6 g, 112 mmol) and PTSA (0.71 g, 4.00 mmol) were added under room temperature and the reaction mixture was allowed to stir for 4 h. Then it was filtered through an amberlyst column, and the eluted solvent was concentrated and was put under 60°C and full vacuum for 2 h to remove the DMP if present. Then, the residue was dissolved in chloroform and precipitated in hexanes, and this procedure was repeated thrice; the precipitate was dried overnight to give (1), a white solid 90% yield (12.01 g). ^{1}H NMR (400 MHz, $CDCl_3$) δ [ppm] 4.17 (m, 2H), 3.69 (m, 2H), 1.44 (d, d, 6H, *J=12 Hz and 16 Hz*), 1.20 (s, 3H).

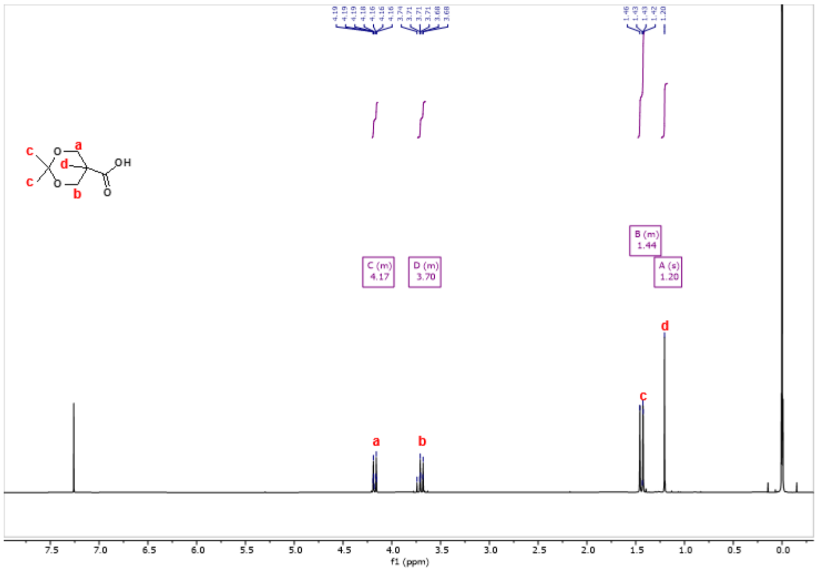

Figure S5. ^{1}H NMR spectrum of 2,2,5-trimethyl-1,3-dioxane-5-carboxylic acid (**1**) in $CDCl_3$.

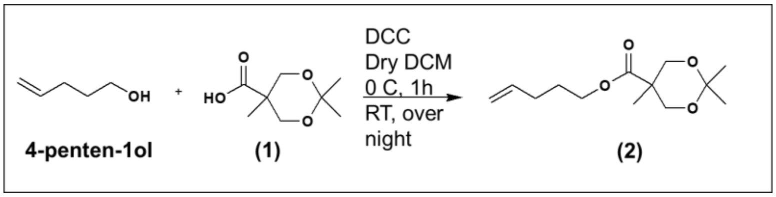

Scheme S4. Synthesis of 4-penten-1-acetonide.

4-penten-1-acetonide (**2**) was synthesized as outlined in **Scheme S4**. 4-penten-1-ol, (0.6 g, 5.8 mmol), 2,2,5-trimethyl-1,3-dioxane-5-carboxylic acid (**1**) (1.9 g, 11.61 mmol), and dimethyl amino pyridine (DMAP) (0.44 g, 3.63 mmol) were stirred in anhydrous DCM (30 mL) at 0°C under argon. DCC (1.2 g, 5.8 mmol) was added to the reaction flask under argon. The reaction mixture was stirred overnight at room temperature. The progression of the reaction was monitored by TLC. Once the reaction reached completion, the reaction mixture was worked up with 0.5 N HCl, concentrated, and the product was then purified via column chromatography using 100% hexanes. (0.85 g, 50% yield) ^{1}H NMR (400 MHz, CDCl3) δ [ppm] 5.80 (m, 1H), 5.02 (m, 2H), 4.18 (t, 2H, *J=11 Hz*), 4.17 (d, 2H, *J=7* Hz), 3.64 (d, 2H, *J=12 Hz*), 2.14 (m, 2H), 1.76 (m, 2H), 1.40 (m, 6H), 1.19 (s, 3H).

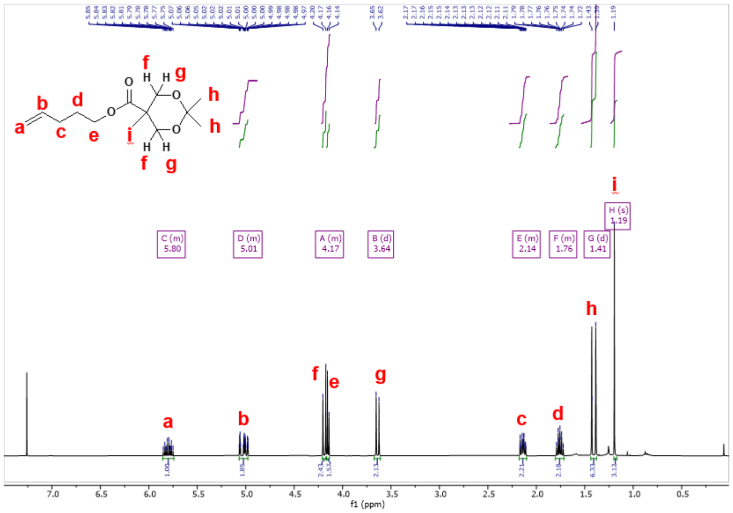

Figure S6. ^{1}H NMR spectrum of 4-pentene-1-acetonide (**2**) in $CDCl_3$.

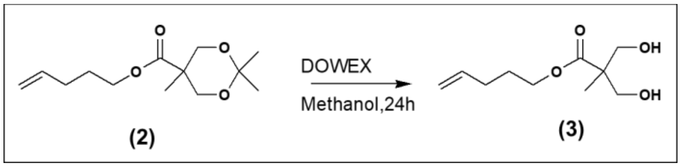

Scheme S5. Synthesis of 4-penten-2-hydroxyl.

4-penten-2-hydroxyl (**3**) was synthesized as outlined in **Scheme S5**. 4-penten-1-acetonide, (**2**) (10.0 g, 35.7 mmol), was dissolved in methanol (200 mL), and 24 g of DOWEX 50W-X2 resin were added to the reaction vessel. The solution was stirred at room temperature for 24 hours and the completion of the reaction was confirmed by ^{1}H NMR. The resin was filtered off and thoroughly washed with methanol. The filtrate was concentrated in vacuum to give **3** (7.56 g, 89%) as a colorless oil. ^{1}H NMR (400 MHz, CDCl3) δ [ppm] 5.73 (m, 1H), 4.96 (m, 2H), 4.12 (t, 2H, *J=8 Hz*), 3.85 (d, 2H, *J=12 Hz*), 3. 65 (d, 2H, *J=12 Hz*), 2.08 (m, 2H), 1.72 (m, 2H), 0.99 (s, 3H).

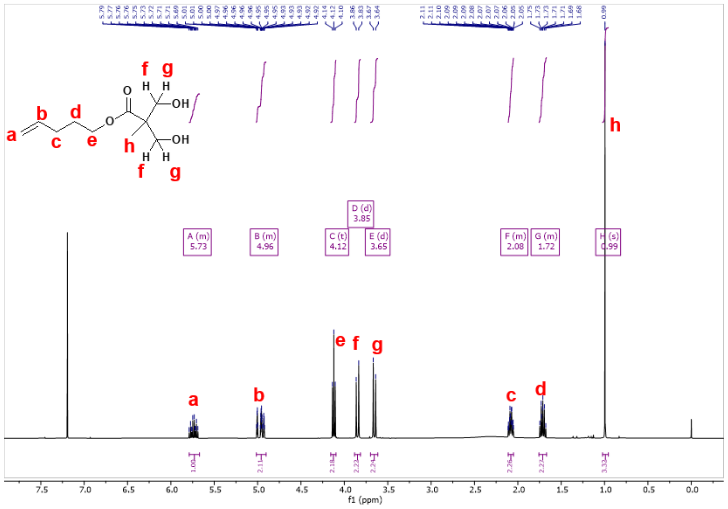

Figure S7. ^{1}H NMR spectrum of 4-pentene-2-hydroxyl (**3**) in $CDCl_3$.

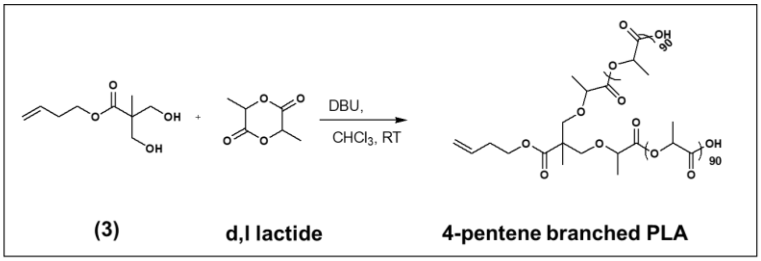

Scheme S6. Polymerization of D, L lactide using 4-penten-2-hydroxyl by ring-opening polymerization.

Branched PLA was synthesized as outlined in **Scheme S6**. The monomer (**3**) (0.050 g, 0.247 mmol) and D, L lactide (3.35 g, 0.24 mmol) were added in a round bottom flask (RBF) sealed with a rubber septum and diluted with anhydrous chloroform. The RBF was subjected to three freeze-thaw cycles before being transferred to the glove box. DBU was purified by maintaining it under vacuum for 7 days and stored under nitrogen before transferring it to the glove box. DBU (0.188 g, 1.236 mmol) was added in another round bottom flask sealed with a rubber septum (subjected to freeze-thaw cycles) maintained in the glove box and diluted with anhydrous chloroform. DBU solution was added dropwise to the monomer solution under argon. The reaction was allowed to stir at room temperature for 4 hours and quenched by adding benzoic acid (0.226 g, 1.85 mmol). The reaction was further allowed to stir for another 1 hour. The resulting viscous liquid was purified from methanol: hexane: ether mixture (1:15:5). The polymer obtained was dissolved in DCM, reprecipitating three times using the above-mentioned mixture. The resulting polymer was finally dried under vacuum for 48 hours.

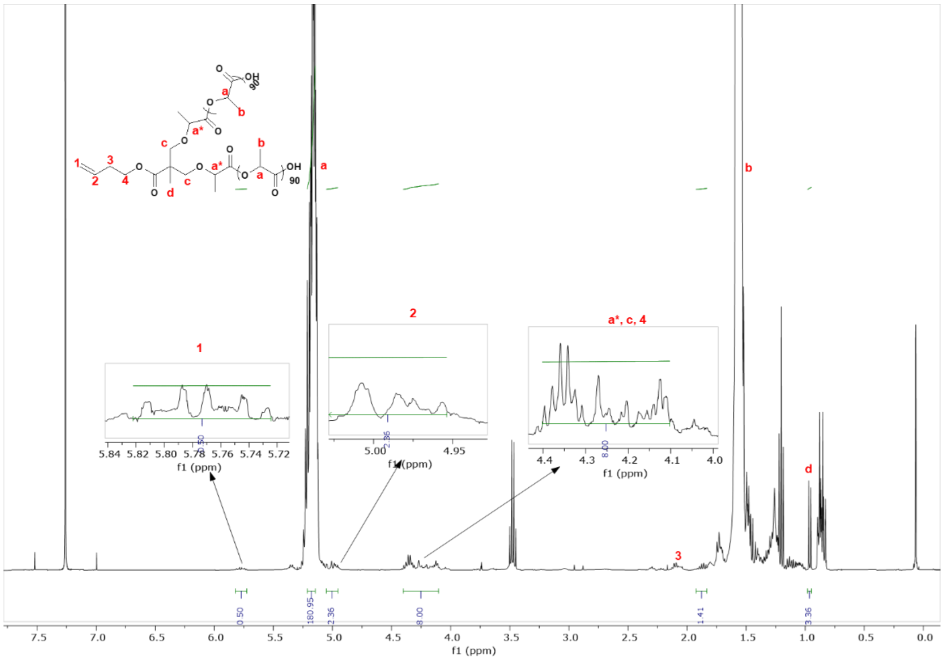

Figure S8. ^{1}H NMR spectrum of branched D, L lactide (**4**) in $CDCl_3$.

1.4 Synthesis of HBCs

The HBCs were synthesized as described in our previous publication,[5] following a thiol-ene photocoupling reaction adapted from the literature was employed.[6-8] Briefly, the hydrophilic polyacrylamide was added in excess to PLA (1.5:1 molar ratio) in order to ensure completion of the reaction, while the ratio of PLA to photoinitiator (DMPA) was kept constant at 1:1. To ensure complete solubilization of starting materials, the hydrophilic polymer was added to DMF and allowed to stir overnight. The following day PLA and the internal standard were added and stirred at 40 °C until completely dissolved. Photoinitiator was then added and the reaction vessel was quickly transferred to a photochemical reactor.

Conversion was monitored using ^{1}H NMR, comparing the disappearance of vinyl protons to the internal standard (**Figure S9)**. After 7 hours, a typical reaction showed coupling conversion of 38% was achieved. Reactions were then kept in dark conditions for 12 hours, and the HBC was purified by precipitation into DI water and filtered to remove unreacted hydrophilic polymer. The solids were then washed with cold THF to remove unreacted PLA. Washed products were then placed under N_2 to remove residual THF and lyophilized to isolate the HBC as a white powder. ^{1}H NMR spectra of starting blocks and coupled product are shown in **Figure S10**. ^{1}H NMR spectra of starting blocks and coupled products display successful incorporation of both blocks. Comparison of integrations at δ [ppm] 5.3 (m, 1H) for PLA and 3.6 (t, 2H) for the polyacrylamides is consistent with 180 repeats in PLA and 2*n repeats for the polyacrylamides (pHEAm, n = 85; pGlcEAm, n = 49; and pGalEAm, n = 53).

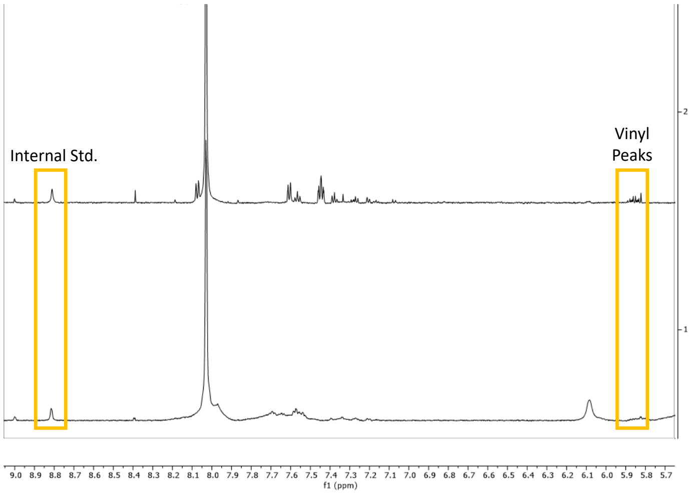

Figure S9. ^{1}H NMR spectra tracking conversion of PLA vinyl bonds during the photocoupling of pGlcEAm with PLA to produce Glc 53:47 in DMF-d_7. Top spectrum is time initial (t0) and bottom spectrum is time final (tf).

A.

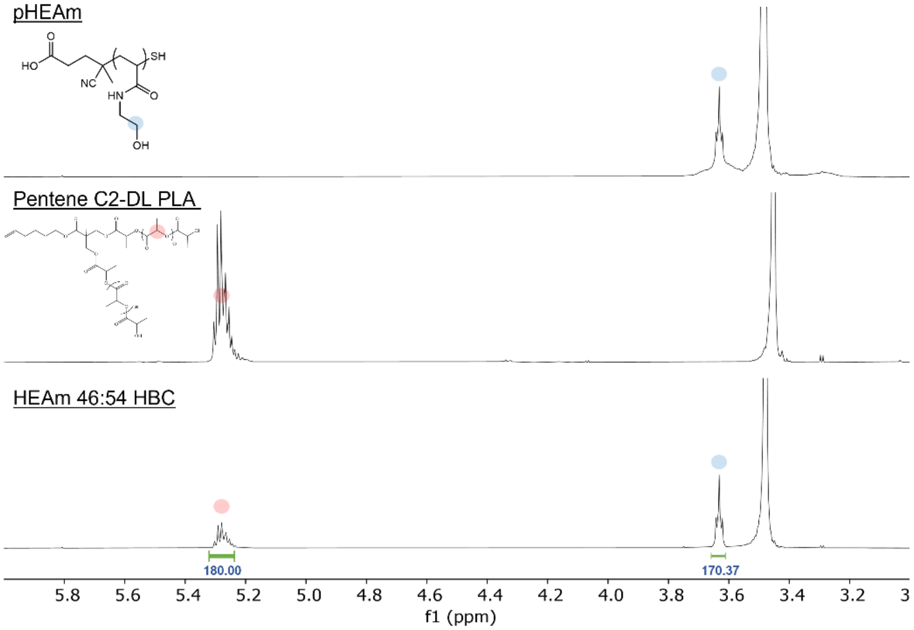

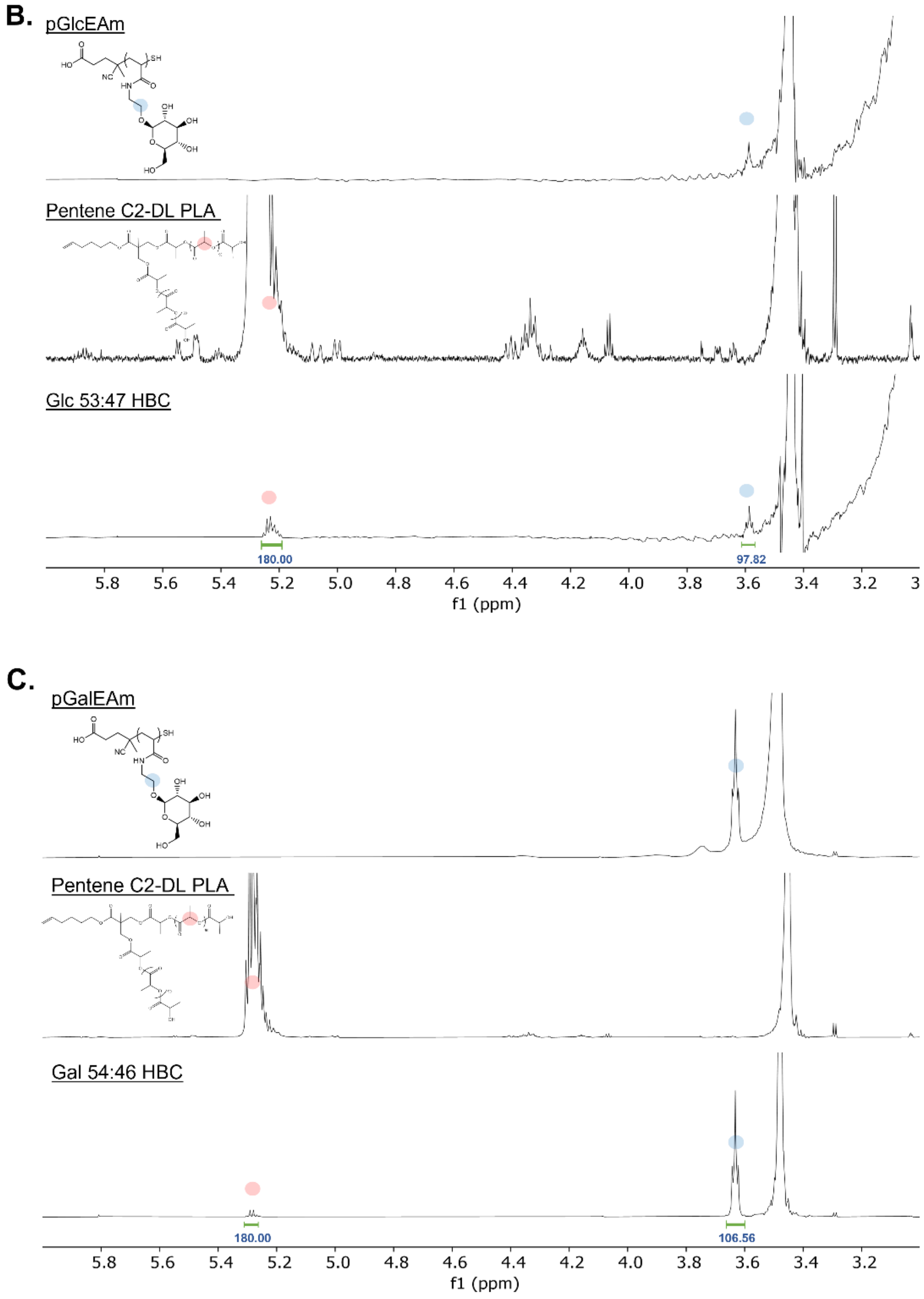

Figure S10. ^{1}H NMR spectra comparison of starting blocks and coupled HBCs A) HEAm 46:54, B) Glc 53:47 and C) Gal 54:46 in DMF-d_7.Integration of the peaks shows successful incorporation of the blocks for approximate 50:50 HHB.

1.5 Encapsulation Studies. Curcumin and methyl orange (MO) dyes were used separately to load into nanoparticles following literature procedures with modifications.[9] The chemical

structures and absorbance profiles for each dye are included in the Supporting Information (**Figure S11**).

For the hydrophobic dye, curcumin (1 mg) and the HBC (1 mg) were dissolved in THF (200 μL). This organic phase was added dropwise to DI water (2 mL) to prepare the final nanoparticle concentration (0.5 mg mL^{-1}). Solutions were then covered with perforated aluminum foil, and the THF was allowed to evaporate overnight before removing by filtration any dye that was not encapsulated and had precipitated from solution. Solutions were then lyophilized, and the resulting powder was then redissolved in THF (2 mL). In order to construct a calibration curve for free curcumin, solutions of dye in THF were prepared with concentrations ranging from 0 to 0.1 mg mL^{-1}, and the absorbance values were determined using a microplate reader to measure absorbance at 430 nm. The dye loading efficiency (DL%) and encapsulation efficiency (EE%) were determined for each nanoparticle composition and dye species using Equations 1 and 2,

$$DL\ (\%) = 100 \times \frac{M_D}{(M_P)} \qquad \text{Equation 1}$$

$$EE\ (\%) = 100 \times \frac{M_D}{(M_{Di})} \qquad \text{Equation 2}$$

with M_D = mass of dye in nanoparticle, M_P = mass of HBC, and M_{Di} = mass of dye initially added during nanoprecipitation.[9]

For hydrophilic dyes (MO), a stock solution in DI water was prepared at 1 mg mL^{-1} to act as the aqueous phase and 2 mg of the HBC was dissolved in THF (200 μL) to act as the organic phase. Similar to the standard nanoprecipitation procedure, the organic phase was added dropwise to the aqueous phase while stirring to prepare the final nanoparticle concentration (0.5 mg mL^{-1}).[10] Solutions were then transferred to 2 kDa MWCO dialysis tubing and dialyzed for 3 days until unencapsulated dye completely escaped, as determined by testing the outer solution by UV-Vis

(absorbance values of 464 nm for MO) until no signal from the dye was detected. The amount of encapsulated dye was calculated using absorbance values compared with a standard calibration curve. To construct the calibration curve, solutions of hydrophilic dye dissolved in DI water were prepared with concentrations ranging from 0 to 0.1 mg mL^{-1}, and a microplate reader was used to measure absorbance at 464 nm. The encapsulated dye, DL (%), and EE (%) were then calculated using Equations 1 and 2.

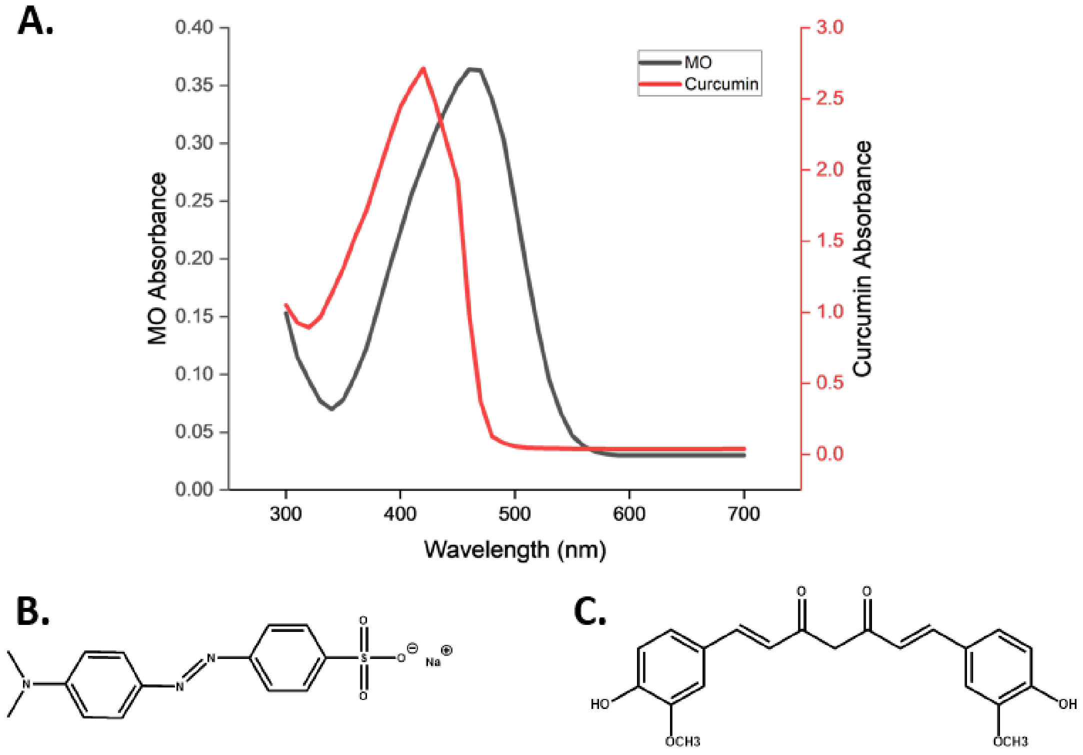

Figure S11. A) Absorbance spectra for dyes used in encapsulation studies, B) and C) structures of MO and curcumin. Absorbance spectra of MO and curcumin were performed in water and THF, respectively.

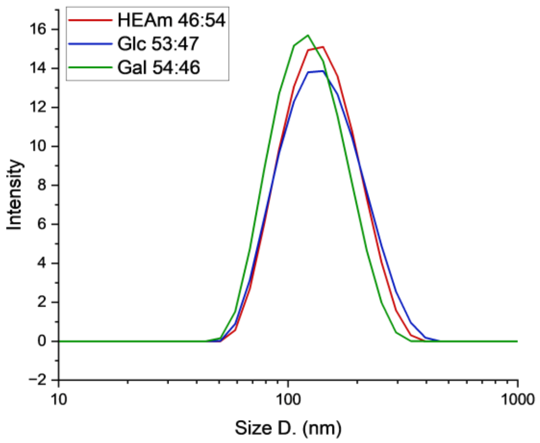

Figure S12. DLS intensity average size distribution of nanoparticles formed in water by nanoprecipitation.

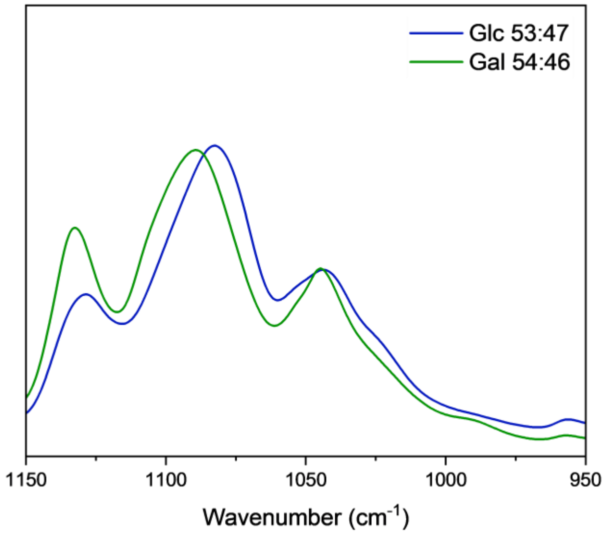

Figure S13. ATR-FTIR overlay of Glc 53:47 and Gal 54:46 spectra highlighting changes in peak shape at 1030 cm^{-1}, indicating a change in hydrogen bonding patterns.

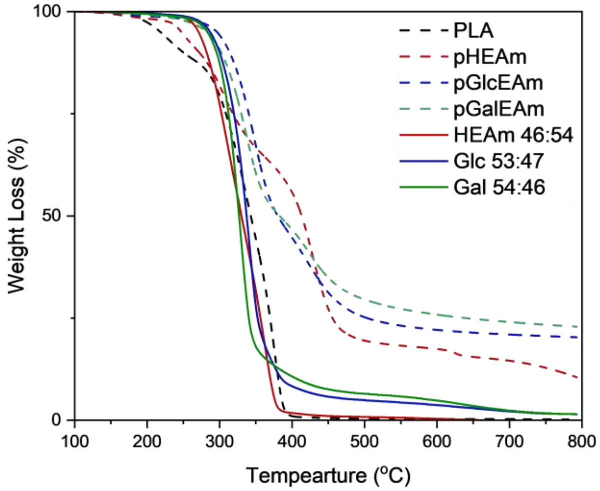

Figure S14. TGA of homopolymer and HBC powders following isothermal hold at 100 °C for 30 minutes.

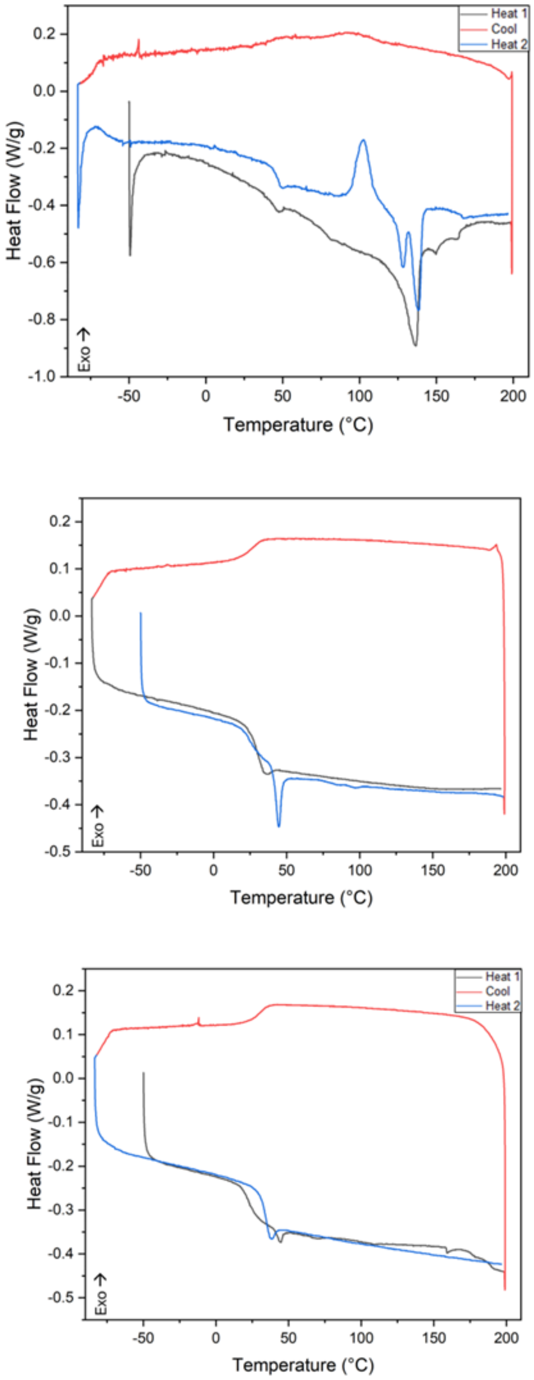

Figure S15. DSC heat-cool-heat cycles. A) Gal 54:48, showing one first order transition on the first heating and multiple on second heating cycle, B) Glc 53:47, showing a crystalline peak on

first heating but only a Tg on second heating, C) HEAm 46:54 showing apparent second order transitions on both heat cycles.

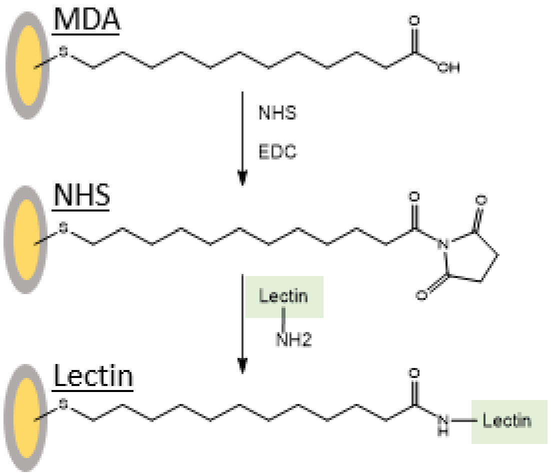

Figure S16. Gold QCM-D sensors grafting process with MDA, NHS, and lectins.

1.6 Cell Viability. Human embryonic kidney cells (HEK293, ATCC) were maintained in Dulbecco's modified eagle's medium (DMEM) supplemented with 10% fetal bovine serum (FBS) and 1% penicillin-streptomycin at 37 °C and 5% CO_2. Cells at 90% confluence were trypsinized (0.25% trypsin ethylenediaminetetraacetic acid (EDTA), 5 min) to dissociate cells, and collected into a falcon tube and centrifuged at 5000 rpm for 5 minutes to pellet. HEK293 cells were resuspended in supplemented DMEM and counted using a hemocytometer to determine cell concentration. Cells were diluted with additional media to a working concentration of 1 x 10^5 cells mL^{-1} and seeded in a 96 well plate (100 µL per well). Seeded cells were left to adhere for 24 hours in an incubator at 37 °C and 5% CO_2. After 24 hours, nanoparticle stock solutions were added to the wells to achieve the desired final concentration with nuclease free water used as a negative control, and Triton X-100 used as positive control for 100 % cytotoxicity (i.e maximum LDH

release). Plates were then incubated for 24 hours at 37 °C and 5% CO_2. Cytotoxicity was evaluated using the CyQUANT LDH Kit (Invitrogen) following manufacturer protocols and was calculated using Equation 3. A microplate reader was used to assess the absorbance at 490 nm with a reference wavelength of 690 nm.

$$\% \text{ Cyto} = \frac{Compound\text{−}treated\ LDH\ Activity - Spontaneous\ LDH\ Activity}{Maximum\ LDH\ Activity - Spontaneous\ LDH\ Activity} * 100 \qquad \text{Equation 3}$$

$$\text{LDH Activity} = \frac{\text{Abs.Sample}_{490} - \text{Abs.Sample}_{690}}{\text{Abs.MLDH}_{490} - \text{Abs.MLDH}_{690}} * 100 \qquad \text{Equation 4}$$

1.7 Live/Dead Imaging. The LIVE/DEAD™ Cell Imaging Kit (Invitrogen) was used to assess cell cytotoxicity at the highest concentration of nanoparticle treatment following the manufacturer's protocols. Cells were plated and treated following the cytotoxicity protocol. Following 24-hour incubation with nanoparticle treatments, 80 µL of media was removed from each well, leaving 20 µL and an equal volume of freshly prepared Live/Dead (Calcein-AM and BOBO-3) stock solution added to each well. This was left to incubate for 15 minutes at room temperature in the dark. Five representative images were then collected per treatment using the Leica DM IL LED Fluo SE inverted fluorescent microscope.

References:

1. Das, P. K.; Dean, D. N.; Fogel, A. L.; Liu, F.; Abel, B. A.; McCormick, C. L.; Kharlampieva, E.; Rangachari, V.; Morgan, S. E., Aqueous RAFT Synthesis of Glycopolymers for Determination of Saccharide Structure and Concentration Effects on Amyloid beta Aggregation. *Biomacromolecules* **2017,** *18* (10), 3359-3366.
2. Bristol, A. N.; Saha, J.; George, H. E.; Das, P. K.; Kemp, L. K.; Jarrett, W. L.; Rangachari, V.; Morgan, S. E., Effects of Stereochemistry and Hydrogen Bonding on Glycopolymer-Amyloid-beta Interactions. *Biomacromolecules* **2020,** *21* (10), 4280-4293.
3. Stockmal, K. A.; Downs, L. P.; Davis, A. N.; Kemp, L. K.; Karim, S.; Morgan, S. E., Cationic Glycopolyelectrolytes for RNA Interference in Tick Cells. *Biomacromolecules* **2022,** *23* (1), 34-46.
4. Yaddehige, M. L.; Chandrasiri, I.; Barker, A.; Kotha, A. K.; Dal Williams, J. S.; Simms, B.; Kucheryavy, P.; Abebe, D. G.; Chougule, M. B.; Watkins, D. L., Structural and Surface Properties of Polyamidoamine (PAMAM) – Fatty Acid-based Nanoaggregates Derived from Self-assembling Janus Dendrimers. *ChemNanoMat* **2020,** *6* (12), 1833-1842.
5. Green, K. A.; Kulkarni, A. S.; Jankoski, P. E.; Newton, T. B.; Derbigny, B.; Clemons, T. C.; Watkins, D. L.; Morgan, S. E., Biocompatible glycopolymer-PLA amphiphilic hybrid block copolymers with unique self-assembly, uptake, and degradation properties. *Biomacromolecules* **2024,** *10.1021/acs.biomac.4c00885*.
6. Wang, Y.; Chou, D. H., A Thiol-Ene Coupling Approach to Native Peptide Stapling and Macrocyclization. *Angew. Chem. Int. Ed. Engl.* **2015,** *54* (37), 10931-4.
7. Hoyle, C. E.; Bowman, C. N., Thiol-ene click chemistry. *Angew. Chem. Int. Ed. Engl.* **2010,** *49* (9), 1540-73.
8. Fairbanks, B. D.; Love, D. M.; Bowman, C. N., Efficient Polymer-Polymer Conjugation via Thiol-ene Click Reaction. *Macromol. Chem. Phys.* **2017,** *218* (18), 1700073.

9. Chandrasiri, I.; Abebe, D. G.; Loku Yaddehige, M.; Williams, J. S. D.; Zia, M. F.; Dorris, A.; Barker, A.; Simms, B. L.; Parker, A.; Vinjamuri, B. P.; Le, N.; Gayton, J. N.; Chougule, M. B.; Hammer, N. I.; Flynt, A.; Delcamp, J. H.; Watkins, D. L., Self-Assembling PCL–PAMAM Linear Dendritic Block Copolymers (LDBCs) for Bioimaging and Phototherapeutic Applications. *ACS Applied Bio Materials* **2020,** *3* (9), 5664-5677.
10. Chandrasiri, I.; Loku Yaddehige, M.; Li, B.; Sun, Y.; Meador, W. E.; Dorris, A.; Farid Zia, M.; Hammer, N. I.; Flynt, A.; Delcamp, J. H.; Davis, E.; Lippert, A.; Watkins, D. L., Cross-linking Poly(caprolactone)–Polyamidoamine Linear Dendritic Block Copolymers for Theranostic Nanomedicine. *ACS Applied Polymer Materials* **2022,** *4* (5), 2972-2986.
